# ﻿Two new and two known species of the genus *Eudorylaimus* Andrássy, 1959 (Nematoda, Dorylaimoidea, Qudsianematidae) from Northeast China

**DOI:** 10.3897/zookeys.1238.138550

**Published:** 2025-05-09

**Authors:** Siwei Liang, Md Niraul Islam, Xiaofang Du, Ying bin Li, Wenju Liang, Wasim Ahmad, Xiaoke Zhang, Mohammad Mahamood, Saleh Alhewairini, Qi Li

**Affiliations:** 1 Tillage and Cultivation Research Institute, Liaoning Academy of Agricultural Sciences, Shenyang-110161, China Tillage and Cultivation Research Institute, Liaoning Academy of Agricultural Sciences Shenyang China; 2 Institute of Applied Ecology, Chinese Academy of Sciences, Shenyang-110016, China Institute of Applied Ecology, Chinese Academy of Sciences Shenyang China; 3 Nematode Biodiversity Research Lab, Department of Zoology, Aligarh Muslim University, Aligarh- 202002, India Aligarh Muslim University Aligarh India; 4 Department of Plant Protection, College of Agriculture and Food, Qasim University, Buraydah, Saudi Arabia Qasim University Buraydah Saudi Arabia

**Keywords:** China, nematode biodiversity, new species, Qudsianematidae

## Abstract

Two new and two known species of the genus *Eudorylaimus* belonging to the family Qudsianematidae are described and illustrated from Northeast China. *Eudorylaimusblisterocaudatus***sp. nov.** is characterized by its 1.0–1.3 mm long body, lip region offset by constriction and 15–17 µm wide; odontostyle 1.0–1.2 times the lip region diameter long, odontophore 1.3–1.5 times the odontostyle length, pharyngeal expansion occupying ~ 41–47% of total neck length; *V* = 59–63%, uterus tripartite 1.3–3.8 times the corresponding body diameter, vulva post-equatorial, spicules 39–45 µm long, 6–8 irregularly spaced ventromedian supplements, tail conoid, sub-digitate with rounded terminus in both the sexes. *Eudorylaimussaccatus***sp. nov.** is characterized by its 1.1–1.4 mm long body, lip region offset by constriction and 15.0–17.5 µm wide; odontostyle 1.0–1.2 times the lip region diameter long, odontophore 1.3–1.5 times the odontostyle length, pharyngeal expansion occupying ~ 44–48% of total neck length; uterus tripartite, 1.3–2.1 times the corresponding body diameter, *V* = 62–63.3%; tail conoid, dorsally convex, with sub-digitate terminus in both the sexes and males with 44–48 µm long spicules and 9–11 irregularly spaced ventromedian supplements. Two known species *E.meridionalis* and *E.caudatus* are reported for the first time from China and one male specimen of *E.caudatus* is recorded here for the first time.

## ﻿Introduction

China is the third largest country in the globe after Russia and Canada. A wide diversity of species can be found in great abundance throughout China’s large and diversified geography. Nematodes are some of the most successful metazoans on Earth (Pilon 2005). They play a crucial role in both terrestrial and aquatic settings (both freshwater and marine), by contributing significantly to ecosystem services (McSorley 2003), and from the top of the mountains to the deep sea, they have colonised every habitat (Schratzberger et al. 2019). In soil, the nematodes of order Dorylaimida Pearse, 1942 occur more frequently and are dominant (Jairajpuri and Ahmad 1992; Peña-Santiago 2021). During the last two decades, various soil ecological and nematological researchers have recorded the dorylaimid nematofauna from the agricultural fields, steppe-forest ecotones, temperate steppes, subtropical forests, grasslands, sandy and black soil in China either to assess the soil health and quality under different treatments of cropland or to assess nematode diversity and community composition of various ecosystems (Jiang et al. 2007; Hu and Qi 2010; Zhang et al. 2010; Pan et al. 2012; Zhong et al. 2016; Hu et al. 2017; Li et al. 2017; Du et al. 2020; Xiong et al. 2021; Li et al. 2023). In contrast, very few authors have made a systematic study of dorylaimid nematode fauna in China. Li et al. (2008) for the first time described four new species: *Tylencholaimusorientalis*, *T.sinensis*, *Proleptonchussinensis*, *Dorylaimoidesalpinus*, and two known species *D.reyesi* Ahmad et al. 2003 and *Basirotyleptuspini* Siddiqi & Khan, 1965 from South-western China. Later, Zhang et al. (2012) recorded a new and three known species of the genus *Tylencholaimellus* N.A. Cobb in M.V. Cobb, 1915 from Changbai Mountain. Wu et al. (2016, 2017a, b, 2018a, b, 2019,) in a series of publications described some new and known species of dorylaimid nematode viz., *Belondirabagongshanensis*, *Labronemellamajor*, *Tylencholaimushelanensis*, *T.ibericus*, *T.cynodonti*, and *Dorylaimoidesshapotouensis*.

*Eudorylaimus* Andrássy, 1959 is one of the most speciose, frequently occurring, widely distributed (Andrássy 2009a) omnivorous, soil-inhabiting nematode taxon belonging to the family Qudsianematidae Jairajpuri, 1965 of the superfamily Dorylaimoidea de Man, 1876 (Dorylaimida). Andrássy (1959) selected *Dorylaimuscarteri* Bastian, 1865 as the type species. After the proposal of the genus, many nematologists added new species until 2022 (Gusakov et al. 2022) but commendable work was done by Tjepkema et al. (1971) and Andrássy (1986, 1991, 2009a, b). Tjepkema et al. (1971) revised the genus dividing it into six species groups (*carteri*, *humulis*, *lugdunensis*, *miser*, *granuliferus*, and *nothus*), and added ten new and redescribed several known species from Indiana. Andrássy (1986) did not accept their proposal on the division of the genus and proposed three more new genera *Epidorylaimus*, *Allodorylaimus*, and *Microdorylaimus* to accommodate some species of *Eudorylaimus* (*E.lugdunensis*, *E.uniformis*, and *E.parvus*). Andrássy (1986, 1991) provided a list of valid species, a list of species that had been transferred to other genera and also provided keys to species of this speciose taxon for the first time. During the last three decades, many authors have described several new species of the genus and recently, Gusakov et al. (2022) described two new species from a lake in Russia. To date, more than 111 species have been recorded worldwide in the genus (Peña-Santigo 2021; Gusakov et al. 2022); of these, only one species, *Eudorylaimuspiceae*Wu et al., 2018a, has been recorded so far from China.

During the nematological surveys in 2019–2020 in Liaoning province of northeast China exploring the dorylaimids of this region, several populations of *Eudorylaimus* were recorded. On a detailed taxonomic study, they were found to represent two known and two new species which are described and illustrated in this paper.

**Figure 1.**
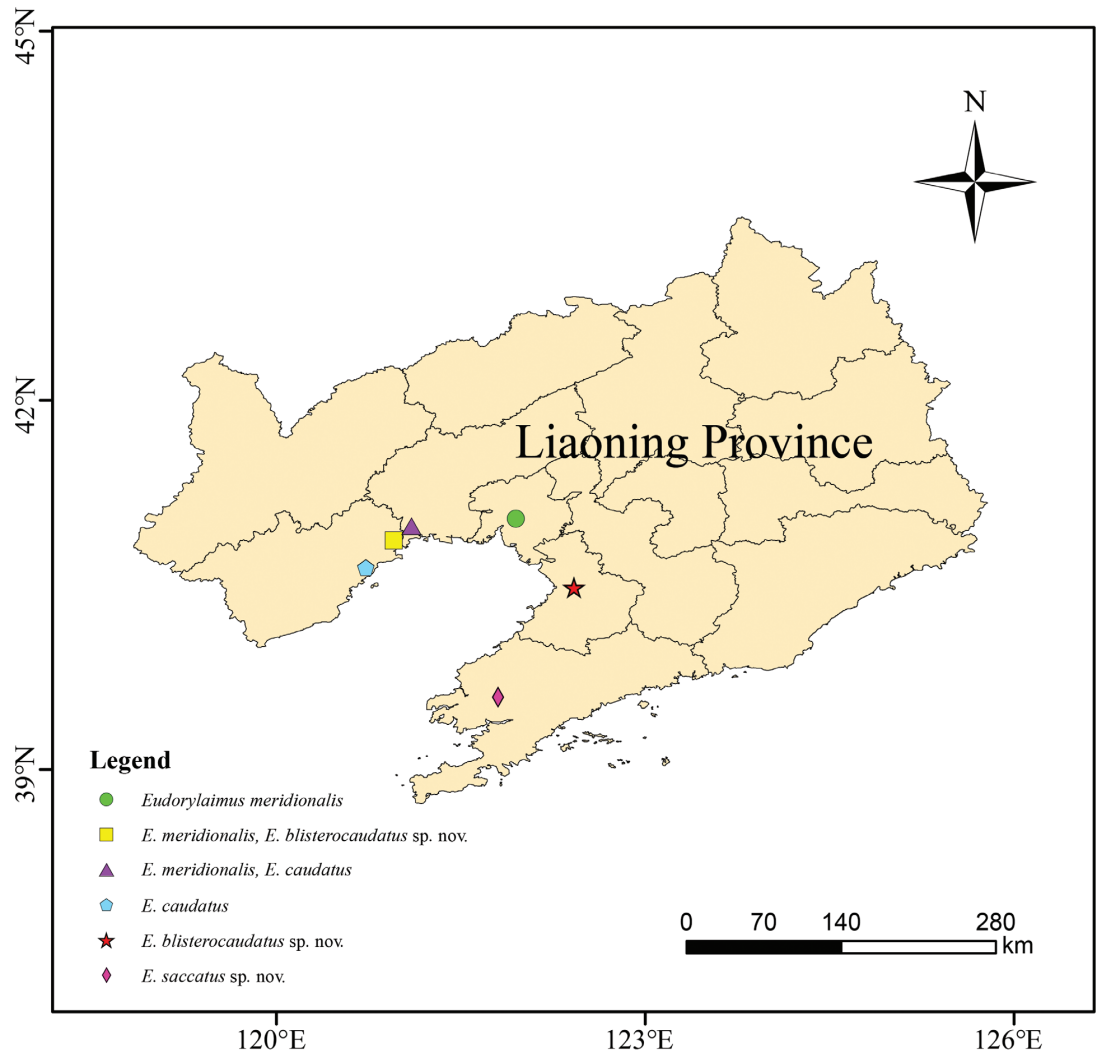
Distribution of *Eudorylaimus* species in the Liaoning province.

## ﻿Materials and methods

### ﻿Site description and sampling

The research survey was conducted in the Liaoning Province of northeast China, situated within a longitude range from 122°14′E to 123°50′E and a latitude range from 39°36′N to 43°27′N. The sampling areas were comparatively level plains. The National Meteorological Information Centre reported that the mean annual temperature (MAT) and mean annual precipitation (MAP) varied between 4.6 °C and 23.1 °C and between 420 mm and 1815 mm, respectively (http://data.cma.cn). Alfisols were the primary soil types in the sampling area, as per the US soil taxonomy (https://websoilsurvey.sc.egov.usda.gov/). A variety of temperature zones and soil types are found in China’s Liaoning areas, which are thought to be generally adequate for identifying the distribution patterns of soil microfauna diversity across latitudinal gradients. Therefore, soil samples were collected from the rhizosphere of grasses (unidentified), wheat (*Triticum* spp. L.), and Poplar (*Populus* spp.) trees of different sampling sites. Using an auger with a diameter of 2.5 cm, soil samples were randomly taken from depths of 0–20 cm, and 5–6 augers of soil were mixed uniformly to make a composite 500 g sample from each location. The fresh soil samples were brought to the laboratory and maintained at 4 °C in a refrigerator for soil nematode extraction.

### ﻿Nematode extraction and processing and LM study

The nematodes were extracted from soil followed by Cobb’s (1918) sieving and decantation and modified Baermann’s funnel methods. Then the specimens were fixed in hot triethanolamine-glycerol fixative and transferred to a glycerine-alcohol solution in a small cavity block which was then kept in a desiccator containing anhydrous calcium chloride for slow dehydration and mounted in anhydrous glycerine. The paraffin wax ring method was used to make permanent mounts (De Maeseneer and d’Herde 1963). An ocular micrometre was used to take measurements, a drawing tube was used to make line drawings, and an Olympus DP27 digital camera connected to an Olympus BX51 microscope was used to capture photographs. Type and other specimens are deposited in the nematode collection of the Institute Applied Ecology, Chinese Academy of Sciences, Shenyang–110016, China (IAE/NC/EU) as well as in the nematode collection of the Department of Zoology, Aligarh Muslim University, Aligarh–202002, India.

## ﻿Taxonomy

### ﻿ Eudorylaimus meridionalis

Taxon classificationAnimaliaDorylaimidaDorylaimidae

Tjepkema, Ferris & Ferris, 1971

ECCEC694-9A82-5E97-B074-43E89A4E260D

Fig. 2 , Table 1

Eudorylaimus meridionalis Tjepkema, Ferris & Ferris, 1971: 23 (original description).

#### Type specimens.

***Holotype*** • Indiana (slide no. 4/3/68E8) – Pardue Nematode Collection, grassy area, Brown County State Park; ***Paratypes*** • same data as holotype (slide no. 7/22/67C10/2♀, 4/5/ 68C19/1♀, 5/31/68B4/1♀, 8/14/68H7/1♀, 4/2/68F1/1♀, 4/1/ 67A1/2♀, 4/4/68N (2)4/3♀, 10/13/ 68A15/1♀), 4/4/68N (3)1/1♀, 4/3/68EB/2♀). Not examined.

#### Material examined.

China • 2♀ (IAE/NC/EU/*E.meridionalis*/1), Liaoning Province, Panjin City, Dawa; 41.037108°N, 121.948279°E; soil samples collected from around the roots of grasses (unidentified) • 3♀ (IAE/NC/EU/*E.meridionalis*/2–3), Liaoning Province, Huludao City, Xingcheng; 40.860423°N, 120.95638°E; soil samples collected from around the roots of grasses (unidentified) • 2♀ (IAE/NC/EU/*E.meridionalis*/4), Liaoning Province, Jinzhou City, Linghai; 40.974492°N, 121.101014°E; soil samples collected from around the roots of grasses (unidentified).

#### Description.

**Figure 2.**
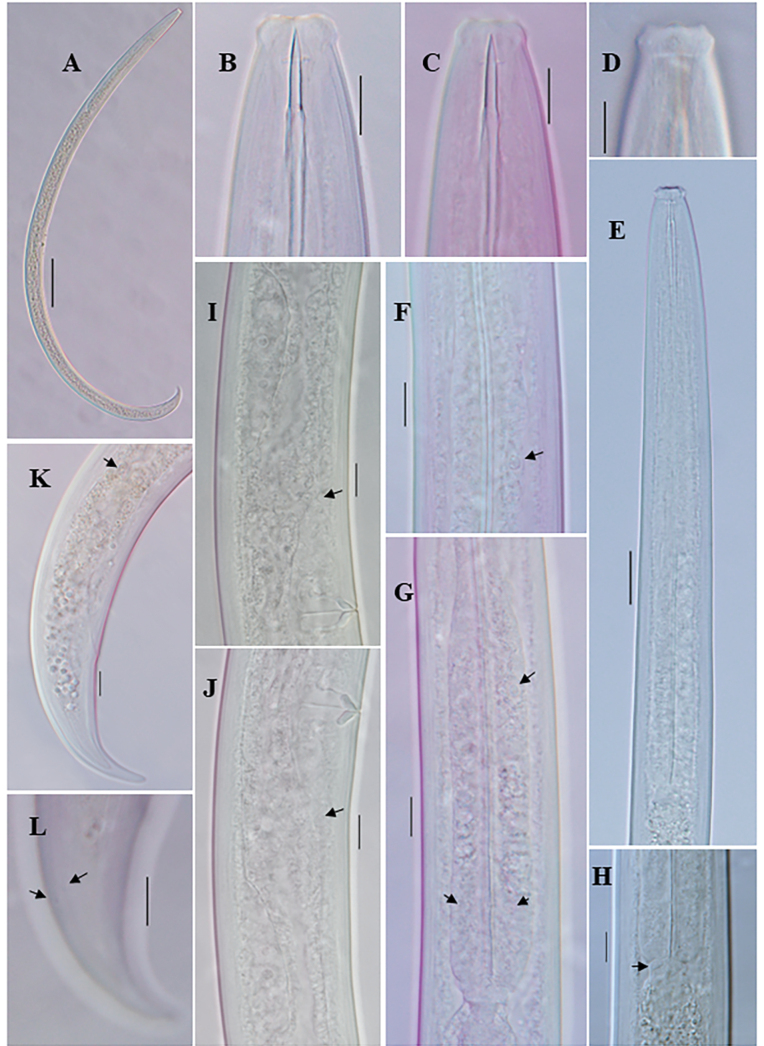
*Eudorylaimusmeridionalis* Tjepkema, Ferris & Ferris, 1971 female: **A** entire **B**, **C** anterior region **D** anterior region showing amphid **E** pharyngeal region **F** pharyngeal expansion (arrow showing DN) **G** expanded part of pharynx (arrow showing DN and S2N) **H** Pharyngo-intestinal junction **I, J** Genital system (arrow showing sphincter) **K** posterior region (arrow showing intestine-prerectum junction). Scale bars: 100 µm (**A**); 10 µm (**B–D**, **F–L**); 20 µm (**E**).

**Female.** Slender nematodes of medium-size, 0.9–1.1 mm long body; curved ventrally or open C-shaped upon fixation. Body cylindrical, tapering gradually towards both extremities but more so towards the posterior region. Cuticle with two distinct layers, 1.5–2.5 μm thick at the anterior region, 2.0–3.0 μm at midbody, and 3.0–4.0 μm on tail. Outer cuticle smooth or finely striated, inner layer with fine transverse striations. Lateral, dorsal, and ventral body pores indistinct. Lateral chords 10–14 μm wide at midbody, occupying ~ 1/3 (29–37%) of the corresponding body diameter. Lip region offset from the body by constriction, 2.5–2.8 times as wide as high or 1/3 to 2/5 (36–43%) of the body diameter at the pharyngeal base. Lips smaller, slightly angular and separated; labial papillae raised from labial contour. Amphidial fovea stirrup-shaped, aperture slit-like, 6.0–7.5 μm wide or occupying 1/2 (46–53%) of lip region diameter. Cheilostom a truncate cone. Odontostyle typical dorylaimoid, 5.2–6.0 times as long as wide, 0.82–1.1 times the lip region diameter long or 1.0–1.5% of total body length, its aperture 4.5–6.0 μm or 1/3 to 2/5 (33–40%) of its length. Odontophore linear, rod-like, 1.3–1.9 times the odontostyle length. Guiding ring simple, at 0.50–0.57 times the lip region diameter from anterior end. Pharynx consisting of a weakly muscular anterior part, expanding gradually into a cylindroid part, occupying ~ 38–42% of the total pharyngeal length; expanded part of the pharynx 5.0–6.2 times as long as wide, 2.8–3.3 times body diameter at neck base. Pharyngeal gland nuclei and their orifices are located as follows: DO = 65.1–67.1, DN = 69.5–71.5, DO–DN = 3.6–5.7, S1N1 = 77.2–79.6, S1N2 = 81.0–83.9, S2N = 90.8–92.8, S2O = 91–93. Nerve ring encircling the pharynx at 38–42% of the neck length from the anterior end. Cardia rounded to conoid, ~ 1/2 (44–58%) of the corresponding body diameter long, with a well-developed cardiac disc. Genital system didelphic–amphidelphic; both the genital branches almost equally developed. Anterior genital branch 7.6–13.6% and the posterior genital branch 9.2–12.3% of the body length. Ovary reflexed, not reaching the oviduct-uterus junction; measuring 45–68 μm or 1.0–2.0 (anterior) and 49–79 μm or 1.1–2.0 (posterior) times the corresponding body diameter long; oocytes arranged in a single row except near the tip. Oviduct joining the ovary sub-terminally, measuring 43–60 μm or 1.1–1.7 (anterior) and 42–66 μm or 1.2–1.6 (posterior) times the corresponding body diameter long; consisting of a slender distal portion and a well-developed ***pars dilatata***. Oviduct-uterus junction marked by a well-developed sphincter. Uterus short, tubular, measuring 31–53 μm or 0.72–1.3 (anterior) and 33–48 μm or 0.88–1.2 (posterior) times the corresponding body diameter long, bipartite, a well-developed muscular proximal part, and a short, slender distal part. Vagina extending inwards, 14–18 μm or ~ 2/5 to 1/2 (39–45%) midbody diameter; ***pars proximalis*** 8–11 × 8.0–10.5 μm, with somewhat sigmoid wall encircled by circular muscles; ***pars refringens*** with two small trapezoid-shaped sclerotized pieces, measuring 5–7 × 2–3 μm and the combined width 7–9 μm; ***pars distalis*** 1.5–2.0 μm. Vulva a transverse slit. Sperm cells absent. Prerectum 1.2–2.4 and rectum 1.0–1.3 times the anal body diameter long. Tail long, conoid, ventrally arcuate with rounded to subacute tip, 1.8–2.3 times anal body diameter long; hyaline part of tail always perceptible, ~ 1/5 to 1/3 anal body diameter long with a pair of caudal (Fig. 2L) pores on dorsal side.

**Table 1.** Morphometrics of *Eudorylaimusmeridionalis* Tjepkema, Ferris & Ferris, 1971. All measurements are in μm and in the form: mean ± s.d. (range).

Localities	Panjin population	Huludao population	Jinzhou population
Characters	Females	Females	Females
*n*	2	3	2
L	1092, 972	1106.3 ± 5.3 (1100–1113)	964, 957
Body diameter at neck base	34, 31	36.3 ± 1.2(35–38)	35, 31
Body diameter at mid body	36, 34	40.8 ± 1.5(39.5–43)	38, 32
Body diameter at anus	23, 22	24.6 ± 0.94(24–26)	22, 23
a	30.3, 28.5	27.13 ± 0.99(25.7–27.8)	25.3, 29.9
b	4.0, 4.0	3.9 ± 0.008(3.95–3.97)	3.7, 3.7
c	23.7, 19.1	23.2 ± 0.97(22.26–24.5)	21.0, 22.3
c’	2.0, 2.3	1.93 ± 0.10(1.8–2.0)	2.1, 1.9
V	51.6, 52.4	51.8 ± 0.71(50.9–52.6)	53.9, 54.3
G1	9.2, 13.6	8.5 ± 0.88(7.6–9.7)	10.9, 9.19
G2	10.0, 12.3	9.8 ± 0.06(9.7–9.9)	11.3, 9.2
Lip region diameter	14, 13	13.6 ± 0.47(13–14)	13, 13.5
Lip region height	5.0, 5.0	5.16 ± 0.23(5.0–5.5)	5.0, 5.0
Amphidial aperture	7.0, 6.0	7.16 ± 0.23(7.0–7.5)	7.0, 7.0
Odontostyle length	14, 13	13.8 ± 1.6(11.5–15)	15, 14
Odontophore length	23.0, 20.5	21.5 ± 0.40(21–22)	20, 19.5
Total stylet length	37, 33.5	35.3 ± 1.3(33.5–36.5)	35, 33.5
Guiding ring from anterior end	7.0, 7.0	7.0	7.5, 7.5
Nerve ring from anterior end	106, 100	116.6 ± 1.2(115–118)	105, 104
Neck length	272, 243	279.3 ± 1.6(277–281)	260, 256
Expanded part of pharynx	104, 101	113.0 ± 4.2(110–119)	106, 102
Cardia length	17, 18	18.6 ± 1.6(17–21)	18, 18
Anterior genital branch	101, 133	95.0 ± 10.1(85–109)	106, 88
Posterior genital branch	110, 120	109 ± 0.81(108–110)	109, 89
Vaginal length	16, 15.5	17.0 ± 0.81(16–18)	15, 14
Vulva from anterior end	564, 509	574 ± 9.9(560–582)	520, 520
Prerectum length	56, 36	50.3 ± 5.9(42–55)	27, 36
Rectum length	30, 28	29.6 ± 1.8(27–31)	27, 24
Tail length	46, 51	47.6 ± 2.0(45–50)	46, 43

**Male.** Not found.

#### Remarks.

Tjepkema et al. (1971) described this species from several localities of Indiana, USA. In the present study, we have recorded this species from several localities of Liaoning Province in China. The present populations are similar to the type population, except that slight morphological and morphometrics variations are observed: odontophore distinct, 1.3–1.9 times odontostyle length (vs odontophore length obscure); comparatively longer cardia (17–21 vs 8–17 μm) and shorter rectum (24–31 vs 45 μm). In the presence of long conoid tail, *E.meridionalis* resembles *E.aquilonarius*Tjepkema et al., 1971 but differs in having comparatively shorter odontostyle (11.5–15 vs 16 μm); differently shaped amphidial aperture (stirrup-shaped vs beaker-shaped) and *pars refringens* (trapezoidal vs triangular shaped) and shorter prerectum (27–56 vs 59 μm). This species is recorded here for the first time from China.

### ﻿ Eudorylaimus caudatus

Taxon classificationAnimaliaDorylaimidaDorylaimidae

Mushtaq & Ahmad, 2006

FFF3A4B9-625F-5F61-B21B-C509ECBFE1DA

Fig. 3 , Table 2

Eudorylaimus caudatus Mushtaq and & Ahmad, 2006: 20–24, figs 6, 7.

#### Type specimens.

***Holotype*** • India (slide no. *E.caudatus* 1)– Kashmir, Verinag; soil samples collected from around the roots of plants (unidentified); ***Paratypes*** • same data as holotype (slide no. *E.caudatus* 2–4). Not examined.

#### Material examined.

China • 5♀/1♂ (IAE/NC/EU/*E.caudatus*/1–4), Liaoning Province, Huludao City, Xingcheng, 40.640209°N, 120.729788°E; soil samples collected from around the roots of Black locust (*Robiniapseudoacacia* L.) • 2♀ (IAE/NC/EU/*E.caudatus*/5), Liaoning Province, Jinzhou City, Linghai; 40.974492°N, 121.101014°E; soil samples collected from around the roots of grasses (unidentified).

#### Description.

**Female.** Slender nematodes of small-size, 0.88–0.93 mm long body; curved ventrally or open C-shaped upon fixation. Body cylindrical, tapering gradually towards both extremities but more so towards the posterior region. Cuticle with two distinct layers, 1.5–2.0 μm thick at the anterior region, 2.0–3.5 μm at midbody, and 3.0–4.0 μm on tail. Outer cuticle smooth or finely striated, inner layer thin with fine transverse striations. Lateral, dorsal and ventral body pores indistinct. Lateral chords 9–10 μm wide at midbody, occupying ~ 1/3 (27–32%) of the corresponding body diameter. Lip region offset from the body by constriction, 2.0–2.3 times as wide as high or ~ 1/3 (33–37%) of the body diameter at the pharyngeal base. Lips angular, separated; labial papillae slightly raised from labial contour. Amphidial fovea cup-shaped, aperture slit-like, 5.5–6.0 μm wide or occupying ~ 1/2 (50–54%) of lip region diameter. Cheilostom a truncate cone. Odontostyle typical dorylaimoid, 5.0–6.5 times as long as wide, 1.1–1.2 times the lip region diameter long or 1.3–1.5% of total body length, its aperture 5.0–6.0 μm or ~ 2/5 (38–44%) of its length. Odontophore linear, rod-like, 1.2–1.4 times the odontostyle length. Guiding ring simple, at 0.59–0.66 times lip region diameter from anterior end. Pharynx consisting of a weakly muscular anterior part, expanding gradually into a cylindroid part, occupying ~ 40–43% of the total neck length; expanded part of pharynx 5.6–7.1 times as long as wide, 3.5–4.2 times the body diameter at neck base. Pharyngeal gland nuclei and their orifices are located as follows: DO = 60.9–64.1, DN = 64.1–66.8, DO–DN = 2.4–4.1, S1N1 = 76.1–78.3, S1N2 = 80.0–82.5, S2N = 90.0–91.7, S2O = 91.1–92.7. Nerve ring encircling the pharynx at 37–40% of the neck length from the anterior end. Cardia rounded to conoid, ~ 2/5 to 1/2 (37–50%) of the corresponding body diameter long. Genital system didelphic-amphidelphic; both the genital branches almost equally developed. Anterior genital branch 11.4–15.1% and the posterior genital branch 10.3–13.5% of body length. Ovaries reflexed, not reaching the oviduct-uterus junction; measuring 48–87 μm or 1.6–1.9 (anterior) and 49–65 μm or 1.6–1.8 (posterior) times the corresponding body diameter long; oocytes arranged in a single row except near the distal end. Oviduct joining the ovaries sub-terminally, measuring 48–65 μm or 1.6–1.9 (anterior) and 45–82 μm or 1.3–1.8 (posterior) times the corresponding body diameter long, consists of a slender distal portion and a well-developed ***pars dilatata***. Oviduct-uterus junction marked by a well-developed sphincter. Uterus long, measuring 48–58 μm or 1.3–1.8 (anterior) and 40–57 μm or 1.2–1.8 (posterior) times the corresponding body diameter long, tripartite, consists of a well-developed muscular proximal part, a short slender median part, and a short, somewhat spheroid distal part. Vagina cylindrical, extending inwards, 14–16 μm or ~ 2/5 to 1/2 (43–47%) of midbody diameter; ***pars proximalis*** 9.5–12 × 7.0–9.0 μm, with somewhat sigmoid wall encircled by circular muscles; ***pars refringens*** with two small triangular sclerotized pieces, measuring 3–4 × 2–3 μm with the combined width 5–6 μm; ***pars distalis*** 1.5–2.0 μm. Vulva a transverse slit. Prerectum 1.6–2.2 and rectum 0.9–1.1 anal body diameter long. Tail short conoid, ventrally arcuate with clavate to rounded tip, 1.3–1.5 times anal body diameter long, with characteristic series of thickening (blisters) on ventral side; hyaline portion of tail always perceptible, ~ 1/6 to 1/3 anal body diameter long with a pair of caudal pores on dorsal side.

**Figure 3.**
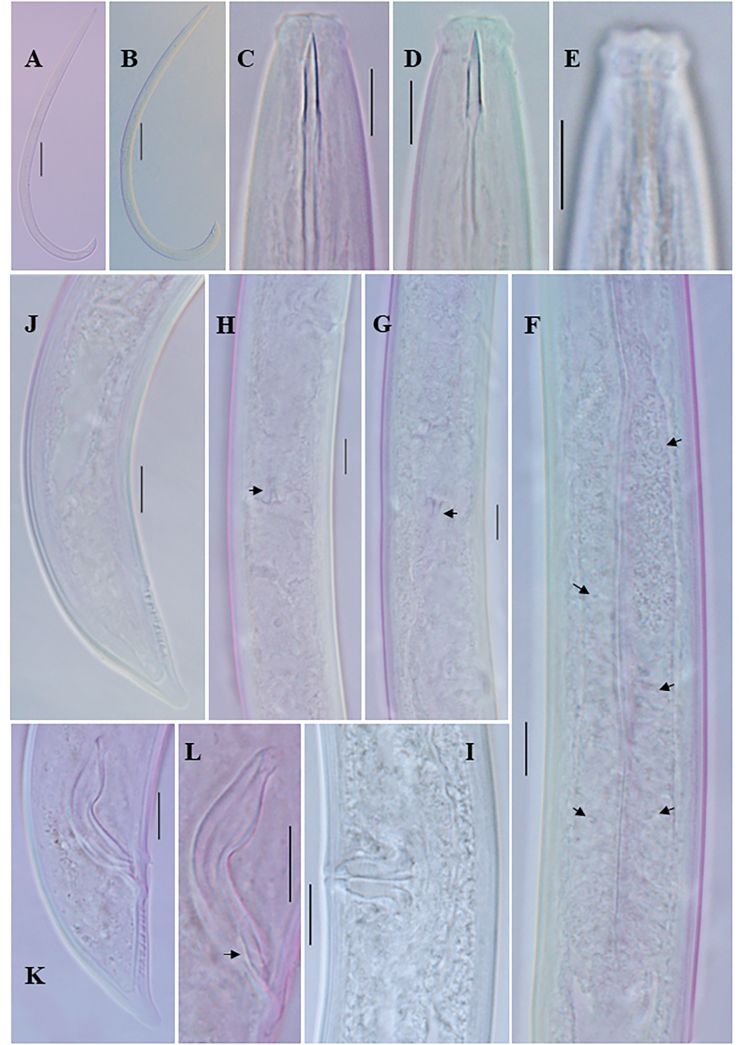
*Eudorylaimuscaudatus* Mushtaq & Ahmad, 2006 **A** entire female **B** entire male **C** anterior region **D** anterior region of male **E** anterior region showing amphid **F** expanded part pharynx **G, H** female genital system **I** vulval region **J** female posterior region **K** male posterior end **L** spicules (arrow showing lateral guiding piece). Scale bars: 100 µm (**A**, **B**); 10 µm (**C–L**).

**Table 2.** Morphometrics of *Eudorylaimuscaudatus* Mushtaq & Ahmad, 2006. All measurements are in μm and in the form: mean ± s.d. (range).

Localities	Huludau population		Jinzhou Population
Characters	Females	Male	Females
*n*	5	1	2
L	920 ± 9.4(902–930)	939	927, 884
Body diameter at neck base	30.2 ± 1.9(29–34)	30	31, 29
Body diameter at mid body	31.4 ± 2.8(29.5–37)	33	33, 32
Body diameter at anus	20.2 ± 0.4(20–21)	19	21, 20.5
a	29.5 ± 2.3(24.9–31.2)	28.4	28.0, 27.7
b	3.2 ± 0.04(3.1–3.2)	3.3	3.1, 3.1
c	31.3 ± 1.0(30.0–32.9)	31.3	28.1, 27.8
c’	1.4 ± 0.06(1.3–1.5)	1.5	1.6, 1.6
V	60.2 ± 0.96(59.0–61.8)	–	60.6, 60.5
G1	13.1 ± 1.1(12.1–15.1)	–	11.4, 13.5
G2	12.3 ± 1.2(10.3–13.5)	–	12.8, 11.8
Lip region diameter	11.1 ± 0.2(11.0–11.5)	12	11, 10.5
Lip region height	5.1 ± 0.2(5.0–5.5)	6	4.5, 5
Amphidial aperture	5.9 ± 0.2(5.5–6.0)	6.5	5.5, 5.5
Odontostyle length	13.2 ± 0.50(12.5–14.0)	14	13, 13
Odontophore length	17.7 ± 0.67(16.5–18.5)	17.5	17.5, 18
Total stylet length	30.9 ± 0.2(30.5–31)	31.5	30.5, 31
Guiding ring from anterior end	6.9 ± 0.2(6.5–7.0)	7.0	7, 7
Nerve ring from anterior end	111.8 ± 3.5(108–117)	112	105, 109
Neck length	286.2 ± 2.3(282–289)	283	281, 291
Expanded part of pharynx	119.2 ± 1.6(117–122)	122	115, 121
Cardia length	9.8 ± 1.3(8.0–12)	13	11, 13
Anterior genital branch	121.2 ± 10.8(112–140)	–	106, 120
Posterior genital branch	113.4 ± 10.7(96–125)	–	105, 119
Vaginal length	14.4 ± 0.8(14–16)	–	15, 15
Vulva from anterior end	554.0 ± 9.7(544–571)	–	562, 537
Prerectum length	38.0 ± 3.6(33–44)	60	34, 33
Rectum length	20.4 ± 1.8(19–24)	36	20, 24
Tail length	29.4 ± 1.0(28–31)	30	33, 32
Spicules length	–	32	–
Lateral guiding pieces	–	10	–
Ventromedian supplements	–	5	–

**Male.** General morphology similar to that of female except for the posterior region being more ventrally curved. Genital system diorchic, testes opposed, sperm cells spindle-shaped, 4.0–5.0 μm long. In addition to the adcloacal pair, situated at 7 µm from the cloacal aperture, there are five irregularly spaced ventromedian supplements, first ventromedian supplement located beyond the range of spicules, 33 μm from the adcloacal pair. Spicules typically dorylaimoid, curved ventrad, relatively robust, 4.9 times as long as wide and 1.6 times as long as body diameter at the level of cloacal aperture, dorsal contour regularly convex, ventral contour bearing a moderately developed hump, curvature of 125°. Head occupying 15.6% of total spicule’s length, its dorsal side longer than ventral side, both sides slightly curved. Median piece 8.7 times as long as wide or occupying ~ 46% of the spicules’ maximum width, reaching the spicule tip, posterior end 2.5 μm wide. Lateral guiding pieces distinct, and rod-like with bifid distal tip, ~ 5.0 times as long as wide or ~ 1/3 of the spicules length. Prerectum 3.2 and rectum 1.9 cloacal body diameter long. Tail similar to female, short conoid, ventrally arcuate with clavate to rounded tip, 1.8 times cloacal body diameter long, with characteristic series of thickening (blisters) on ventral side; hyaline portion of tail always perceptible, 1/3 anal body diameter long with a pair of caudal pores on dorsal side.

#### Remarks.

Mushtaq and Ahmad (2006) described this species from Verinag, Jammu and Kashmir, India. They named this species *E.caudatus* because of the presence of ventral thickenings (blisters) on the tail. In the present study, we have recorded seven females and one male of *E.caudatus* from the Liaoning Province of China. The morphology and morphometric values are similar to the type population except in having slightly longer body size (L = 0.88–0.93 vs 0.79–0.85 mm); longer pharynx (282–291 vs 210–245 μm) and its expansion (115–122 vs 99–113 μm), slightly lower b value (3.1–3.3 vs 3.4–3.7); slightly posteriorly located nerve ring (105–1117 vs 77–87 μm); slightly shorter rectum (19–24 vs 24.5–26.5 μm) and presence of male (vs male absent). These variations should be considered as intraspecific as well as geographical variability. The male is reported here for the first time for this species. This species is recorded here for the first time from China.

### ﻿ Eudorylaimus blisterocaudatus sp. nov.

Taxon classificationAnimaliaDorylaimidaDorylaimidae

89FC6489-3052-5274-9DA4-E2347AF20823

https://zoobank.org/9A3E10B9-2F37-40D0-8289-24485548C249

**Figure 4.**
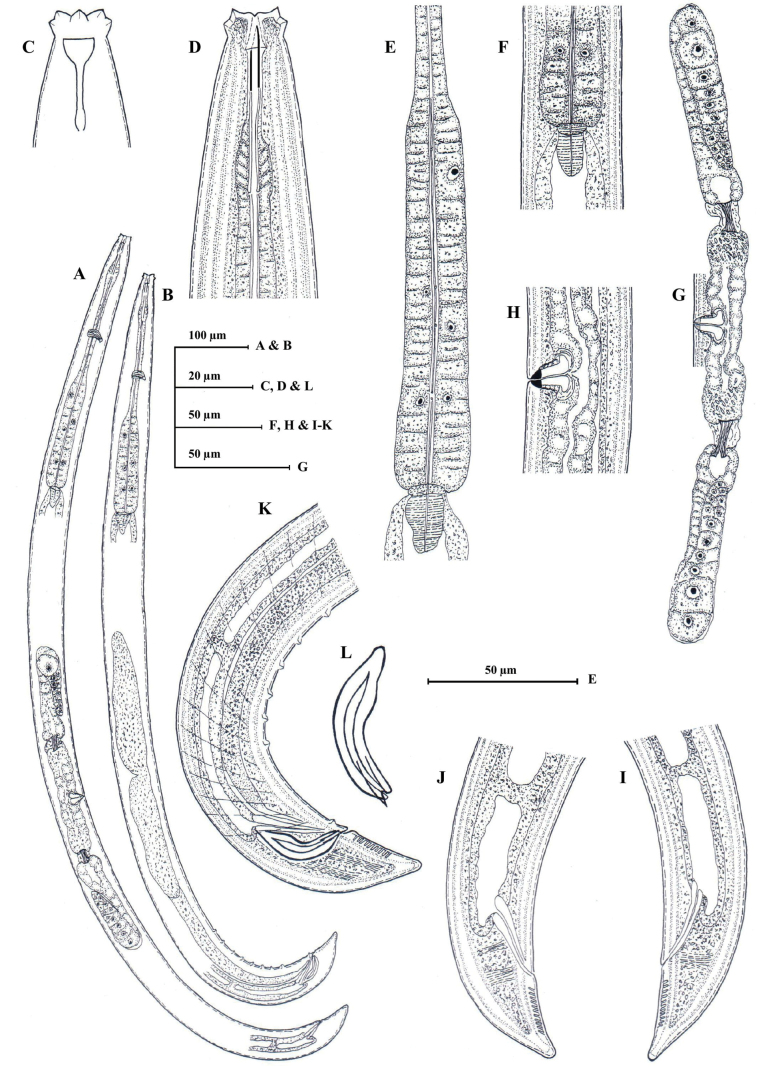
*Eudorylaimusblisterocaudatus* sp. nov. **A** entire female **B** entire male **C** anterior region showing amphid **D** anterior region **E** expanded part pharynx **F** pharyngo-intestinal junction **G** female genital system **H** vulval region **I, J** female posterior region **K** male posterior region **L** spicule.

**Figure 5.**
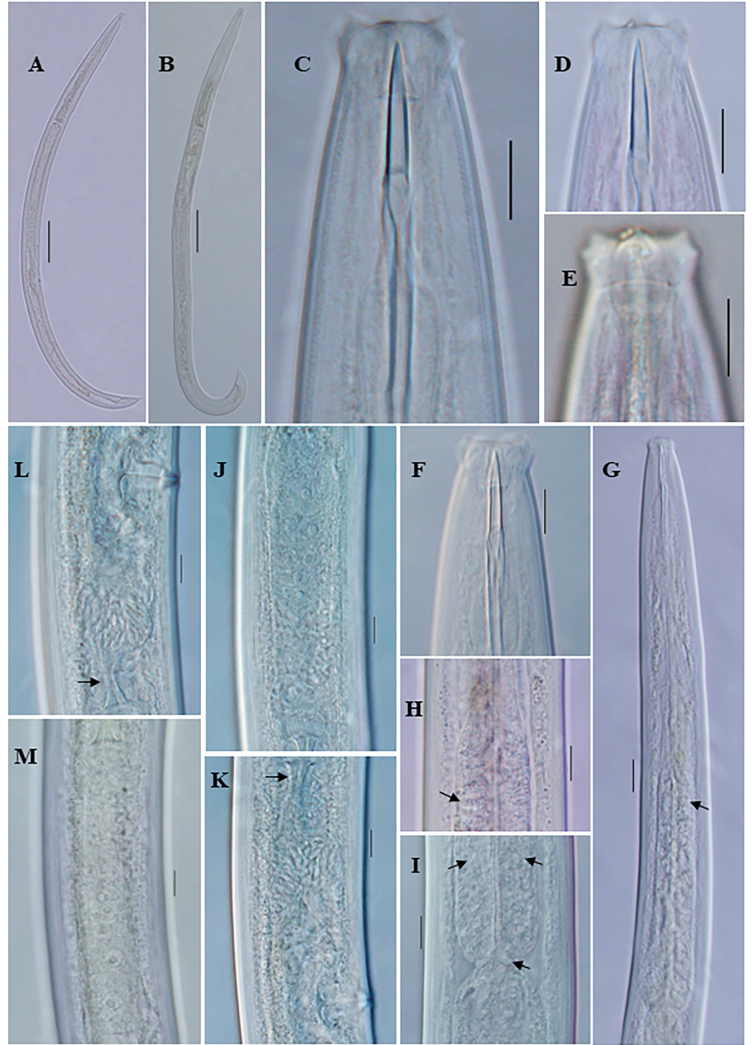
*Eudorylaimusblisterocaudatus* sp. nov. **A** entire female **B** entire male **C, D** female anterior region **E** anterior region showing amphid **F** male anterior region **G** pharyngeal region **H** pharyngeal expansion **I** pharyngo-intestinal junction (arrow showing S2N and cardiac disc) **J, K** anterior genital branch (arrow showing sphincter) **L, M** posterior genital branch (arrow showing sphincter). Scale bars: 100 µm (**A, B**); 10 µm (**C–F**, **H–M**); 20 µm (**G**).

**Figure 6.**
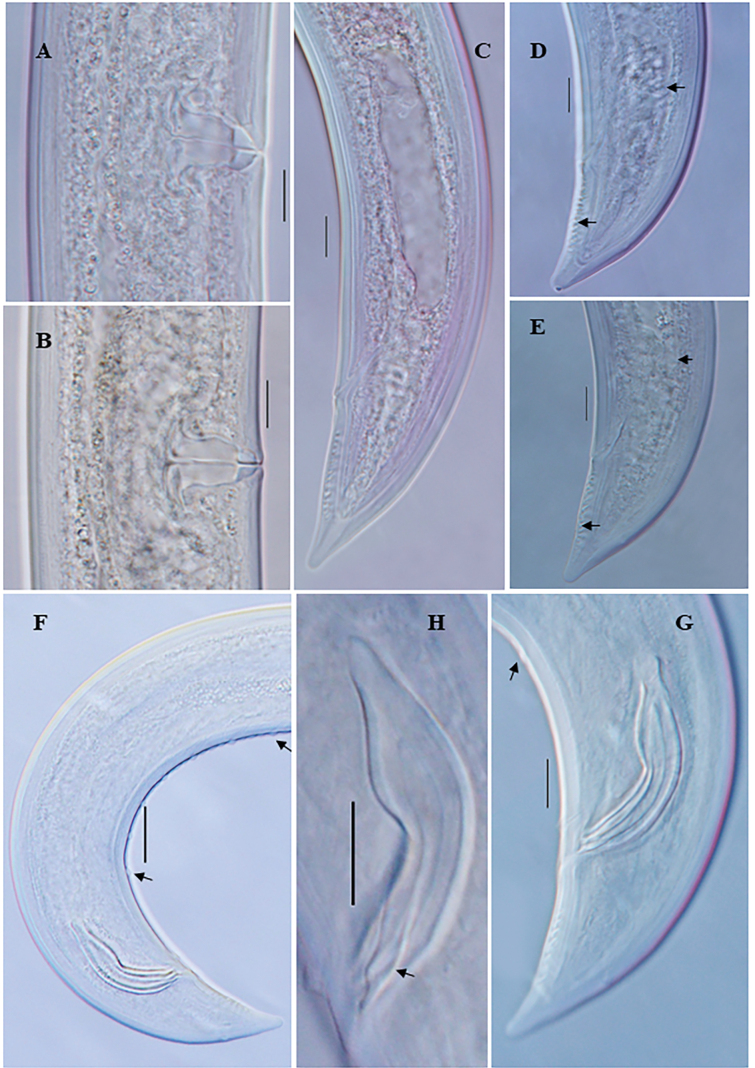
*Eudorylaimusblisterocaudatus* sp. nov. **A, B** vulval region **C** female posterior region **D, E** female posterior end (arrow showing blind sac and blisters) **F** male posterior region **G** male posterior end **H** Spicules. Scale bars: 10 µm (**A–E, G, H)**; 20 µm (**F**).

Figs 4 , 5 , 6 , Tables 3 , 4

#### Type material.

***Holotype*** • China (IAE/NC/EU/*E.blisterocaudatus* /1), Liaoning Province, Huludao City, Xingcheng; 40.860423°N, 120.95638°E; soil samples collected from around the roots of grasses (unidentified). ***Paratypes*** • China (4♀/4♂; IAE/NC/EU/*E.blisterocaudatus*/2-5), same data as holotype. (2♀ IAE/NC/EU/*E.blisterocaudatus*/6), Liaoning Province, Yingkou City, Gaizhou; 40.475832°N, 122.421428°E; soil samples collected from around the roots of Poplar (*Populus* L.).

#### Diagnosis.

*Eudorylaimusblisterocaudatus* sp. nov. is characterized by its 1.0–1.3 mm long slender body; lip region offset by constriction and 15–17 μm broad; odontostyle 18–19 μm with an aperture ~ 37–40% of its length, odontophore 24–28 μm long, 1.3–1.5 times the odontostyle length, total stylet length 42–46 μm; pharynx 300–339 μm long, pharyngeal expansion 129–156 μm or ~ 41–47% of the total pharyngeal length; cardia long 17–26 μm or 0.42–0.58 times the corresponding body diameter long; female genital system didelphic-amphidelphic, uterus long, well-differentiated; V = 59–63%; tail short (35–49 μm, c = 23.3–34.7, c’ = 1.2–1.7), conoid with rounded to sub-clavate terminus, and bearing series of blisters (thickening) on the ventral side, hyaline part 20–33% of its length; males with 39–45 µm long spicules, lateral guiding pieces rod-like, slightly curved ventrally, bifurcated with claw-like distal end, and 6–8 irregularly spaced ventromedian supplements with hiatus.

**Table 3.** Morphometrics of *Eudorylaimusblisterocaudatus* sp. nov. All measurements are in μm and in the form: mean ± s.d. (range).

Characters	Holotype female	Paratype Females	Paratype Males	Other paratype females
n		4	4	2
L	1126	1188.75 ± 95.8 (1061–1319)	1112.5 ± 26.11(1087–1156)	1145, 1309
Body diameter at neck base	44	43.0 ± 3.5(40–49)	42.1 ± 2.1(39–45)	40, 45
Body diameter at mid body	46	45.0 ± 4.1(41–52)	44.0 ± 1.8(41–46)	45, 50
Body diameter at anus	27	28.5 ± 0.86(28–30)	30.0 ± 1.2(29–32)	26, 29
a	24.4	26.6 ± 3.0(22.0–29.9)	25.3 ± 0.72(24.6–26.5)	25.4, 26.1
b	3.7	3.6 ± 0.19(3.5–3.9)	3.5 ± 0.07(3.4–3.6)	3.6, 3.8
c	26.8	29.4 ± 4.6(23.3–34.7)	30.1 ± 1.7(28.3–33.0)	26.6, 31.9
c’	1.6	1.4 ± 0.12(1.3–1.6)	1.2 ± 0.02(1.2–1.3)	1.7, 1.4
V	61.5	62.1 ± 0.94(60.7–63.2)	–	60.2, 59.1
G1	26.8	17.9 ± 4.1(14.3–26.8)	–	14.3, 16.0
G2	26.3	18.0 ± 3.6(15.3–26.3)	–	15.7, 15.8
Lip region diameter	16	15.6 ± 0.41(15–16)	16.1 ± 0.73(15–17)	16, 16
Lip region height	7.0	6.1 ± 0.21(6.0–6.5)	6.7 ± 0.25(6.5–7.0)	6.5, 6.5
Amphidial aperture	8.0	9.0 ± 0.79(8.0–10)	8.6 ± 0.64(8.0–9.5)	8.0, 8.5
Odontostyle length	18.5	18.2 ± 0.25(18–18.5)	18.2 ± 0.43(18–19)	18, 18
Odontophore length	26	24.7 ± 0.75(24–26)	25.1 ± 0.89(24–26)	28, 27
Total stylet length	44.5	43.0 ± 0.70(42–44)	43.3 ± 1.1(42–45)	46, 45
Guiding ring from anterior end	9.5	8.8 ± 0.41(8.5–9.5)	9.3 ± 0.41(9.0–10)	9.0, 9.0
Nerve ring from anterior end	118	114.5 ± 3.6(110–120)	128.7 ± 15.6(111–153)	122, 131
Neck length	302	321.5 ± 11.9(301–331)	314.2 ± 11.5(300–332)	311, 339
Expanded part of pharynx	135	147.0 ± 7.2(138–156)	139.2 ± 8.9(129–150)	132, 148
Cardia length	23	21.5 ± 3.2(17–23)	22.0 ± 1.2(20–23)	17, 23
Anterior genital branch	302	206 ± 43.3(167–275)	–	164, 210
Posterior genital branch	297	204 ± 25.8(176–246)	–	180, 207
Vaginal length	18	19.2 ± 1.9(17–22)	–	17, 20
Vulva from anterior end	692	739 ± 62.9(645–815)	–	689, 773
Prerectum length	65	57.7 ± 17.8(37–85)	90.5 ± 24.9(58–128)	31, 42
Rectum length	27	34.2 ± 1.9(32–37)	43.0 ± 2.1(40–46)	29, 32
Tail length	42	41.4 ± 3.6(37–49)	37.0 ± 1.4(35–39)	43, 41
Spicules length	–	–	40.7 ± 2.4(39–45)	–
Lateral guiding pieces	–	–	13.0 ± 1.2(12–15)	–
Ventromedian supplements	–	–	6–8	–

#### Description.

**Female.** Slender nematodes of medium-sized, 1.0–1.3 mm long body; curved ventrally or open C-shaped upon fixation. Body cylindrical, tapering gradually towards both extremities but more so towards the posterior region. Cuticle with two distinct layers, 1.5–2.0 μm thick at the anterior region, 2.5–4.0 μm at midbody, and 4.0–5.0 μm on tail. Outer cuticle smooth or finely striated, inner layer thin with fine transverse striations. Lateral, dorsal, and ventral body pores indistinct. Lateral chords 13–15 μm at midbody, occupying ~ 1/3 (28–34%) of the corresponding body diameter. Lip region offset from the body by constriction, 2.2–2.6 times as wide as high or ~ 1/3 to 2/5 (32–40%) of the body diameter at the pharyngeal base. Lips angular, separated. Amphidial fovea cup-shaped, aperture slit-like, 8.0–10 μm wide or occupying ~ 1/2 to 3/5 (50–62%) of lip region diameter. Cheilostom a truncate cone. Odontostyle typical dorylaimid in shape, 6.0–7.4 times as long as wide, 1.1–1.2 times the lip region diameter long or 1.3–1.7% of total body length, its aperture 7.0–7.5 μm or ~ 2/5 (37–40%) of its length. Odontophore linear, rod-like, 1.3–1.5 times the odontostyle length. Guiding ring simple, at 0.5–0.6 times lip region diameter from anterior end. Pharynx consisting of a weakly muscular anterior part, expanding gradually into a cylindroid basal part, occupying ~ 41–47% of total neck length; expanded part of the pharynx 5.1–6.6 times as long as wide, 2.8–3.8 times body diameter at neck base. Pharyngeal gland nuclei and their orifices are located as follows: DO = 57–61, DN = 59–65, DO–DN = 2.3–4.2, S1N1 = 71–76, S1N2 = 77–81, S2N = 87–89, S2O = 88–91. Nerve ring encircling the pharynx at 34–39% of neck length from the anterior end. Cardia rounded to conoid, ~ 2/5 to 3/5 (42–58%) of the corresponding body diameter long, its junction with pharyngeal base apparently surrounded by cardiac disc. Genital system didelphic-amphidelphic; both the genital branches almost equally developed. Anterior genital branch 14.3–26.8% and the posterior genital branch 15.3–26.3% of body length. Ovaries reflexed, not reaching the oviduct-uterus junction; measuring 76–105 μm or 1.7–2.5 (anterior) and 82–110 μm or 1.5–2.2 (posterior) times the corresponding body diameter long; oocytes arranged in a single row except near the distal end. Oviduct joining the ovaries sub-terminally, measuring 82–110 μm or 1.5–2.2 (anterior) and 57–98 μm or 1.0–2.3 (posterior) times the corresponding body diameter long; consisting of a slender distal portion and a well-developed ***pars dilatata***. Oviduct-uterus junction marked by the well-developed sphincter. Uterus well-differentiated, tripartite, the proximal part short and well-developed muscular, the median part also short, wider than the proximal part, the distal part comparatively long, somewhat spheroid, measuring 63–170 μm or 1.4–3.8 (anterior) and 65–124 μm or 1.3–2.8 (posterior) times the corresponding body diameter long. Vagina extending inwards, 17–22 μm or ~ 2/5 to 1/2 (38–46%) midbody diameter; ***pars proximalis*** 12–15 × 8.0–11 μm, with somewhat sigmoid wall encircled by circular muscles; ***pars refringens*** with two small triangular-shaped sclerotized pieces, measuring 5–6 × 3–4 μm and the combined width 7.5–9 μm; ***pars distalis*** 2.0–3.0 μm. Vulva a transverse slit. Sperm cells present (*n* = 4). Prerectum 1.1–3.0 and rectum 1.0–1.3 times the anal body diameter long, a distinct blind sac extending posteriorly to the prerectum-rectum junction (Fig. 6D, E). Tail short, dorsally convex, conoid, almost straight or distal part slightly bent ventrally with a rounded to sub-clavate terminus, 1.3–1.6 times anal body diameter long; with characteristic series of thickening (blisters) on the ventral side of tail (Fig. 6C–E, G); hyaline portion of tail perceptible, ~ 1/5 to 1/3 of total tail length with a pair of caudal pores on dorsal side.

**Table 4.** Some important morphometrics and morphological characters of *Eudorylaimusblisterocaudatus* sp. nov. and its six close species, those having blisters on the ventral side of the tail. All measurements are in um (except for ‘L’ in mm).

Characters	*Eudorylaimusblisterocaudatus* sp. nov.	*E.caudatus* Mushtaq & Ahmad, 2006	*E.pectinus* Mukhina, 1970	*E.pussulosus* Andrássy, 1991	*E.coniceps* Loof, 1975	*E.schraederi* Altherr, 1974	*E.maritus* Andrássy, 1959
*n*	7♀♀/4 ♂♂	13♀♀/1♂	1♀/1♂	?♀♀/?♂♂	17♀♀/16♂♂	?♀♀	?♀♀/?♂♂
L	1.0–1.3/1.0–1.1	0.79–0.93/0.99	1.2/1.1	1.1–1.2/0.96–1.2	1.6–2.6/1.9–2.5	1.6–2.6	2.0–2.3/2.0–2.2
a	22.0–29.9/24.6–26.5	24–31/28.4	26/30	23–28/22–23	29–42/35–43	44–53	30–37/35–37
b	3.5–3.9/3.4–3.6	3.1–3.7/3.3	3.6/3.5	3.8–4.6/3.6–3.8	3.7–5.2/4.3–5.0	4.4	4.0–5.0/3.7–4.7
c	23.3–34.7/28.3–33.0	25–32/31.3	34/32	30–35/25–32	41–54/37–48	31–32	40–43/44–55
c’	1.3–1.7/1.2–1.3	1.2–1.6/1.5	–	1.4/1.3–1.4	1.2–1.6/1.1–1.5	4.4	1.3–1.5/1.2–1.4
Vulval position (V%)	59.1–63.2	59.0–63.6	60.6	48–55	42–49	51	48–50
Lip region	offset by constriction	offset by constriction	offset by constriction	offset by constriction	offset by depression	offset by constriction	offset by depression
Lips shape	Lips distinctly angular, strongly separated	Lips angular, moderately separated	Lips angular, separated	Lips angular, moderately separated	Lips rounded, amalgamated	Lips angular, moderately separated	Lips slightly angular, amalgamated
Amphidial aperture	8.0–10/8.0–9.5	5.0–6.0/6.5	–	–	–	–	–
Odontostyle length	18–18.5/ 18–19	11–14/14	18	20	–	20	24–28
Odontophore length	24–26/24–26	16.5–18.5/17.5	–		–	–	–
Total stylet length	42–44/42–45	28.5–31/31.5	–	–	–	–	–
Neck length	301–339/300–332	210–291/283	–	–	–	–	560–620
Expanded part of pharynx	132–156/129–150	99–121/122	–	–	–	–	–
Expanded part of pharynx (%)	41–47	39–43	–	About 50%	46–52	–	–
Cardia length	17–23/20–23	8.0–14.5/13	–	–	–	–	–
Prerectum length	37–85/58–128	33–52/60	–	–	–	–	–
Tail length	37–49/35–39	25–33	–	–	–	–	–
Tail shape	Conoid, dorsally convex, tip rounded	Conoid, tip rounded	Beak-shaped, tip acute	Dorsally concave, straight to sub-digitate	Conoid, pointed tip	–	Conical, pointed tip
Spicules	39–45	32	32	47–49	60–80	–	70–77
Ventromedian supplements	6–8	5	unknown	17	7–11	–	8–10
Blisters on tail	Present	Present	Absent	Present	Present	Present	Present
Pre-rectal blind sac	Present	Imperceptible	Absent	Absent	Absent	Absent	Absent

**Male.** General morphology similar to that of the female except for the posterior region being more ventrally curved. Genital system diorchic, testes opposed, sperm cells spindle-shaped, 6.0–8.0 μm long. In addition to the adcloacal pair, situated at 7–8 µm from the cloacal aperture, there are six to eight irregularly spaced ventromedian supplements, the first ventromedian supplement located outside the range of spicules, 35–51 μm from the adcloacal pair. Spicules typically dorylaimoid, curved ventrad, relatively robust, 4.3–5.0 times as long as wide and 1.2–1.5 times as long as body diameter at the level of cloacal aperture, dorsal contour regularly convex, ventral contour bearing a moderately developed hump, curvature of 120–127°. Head occupying 16–21% of total spicules length, its dorsal side longer than ventral side, both sides slightly curved. Median pieces 9.6–10 times as long as wide or occupying ~ 33–38% of the spicules’ maximum width, reaching the spicule tip, posterior end 3–4 μm wide. Lateral guiding pieces distinct, rod-like, slightly curved ventrally, bifurcated with claw-like distal end, ~ 4.8–6.0 times as long as wide or ~ 1/3 (30–33%) of the spicules’ length. Prerectum 1.9–4.4 and rectum 1.3–1.5 times the cloacal body diameter long. Tail short, dorsally convex, conoid, almost straight or distal part slightly bent ventrally with a rounded to sub-clavate terminus, 1.3–1.5 times anal body diameter long; with characteristic series of thickening (blisters) on ventral side; hyaline portion of tail perceptible, ~ 1/3 of total tail length long with a pair of caudal pores on dorsal side.

#### Etymology.

The new species is named *Eudorylaimusblisterocaudatus* because of the presence of blisters in the caudal region.

#### Remarks.

With the presence of blisters on tail, the new species is morphologically close to *E.caudatus* Mushtaq & Ahmad, 2006; *E.pectinatus* Mukhina, 1970; *E.pussulosus* Andrássy, 1991; *E.coniceps* Loof, 1975 and *E.schraederi* Altherr, 1974 but differs from former in having a longer body size (L = 1.0–1.3 vs 0.79–0.93 mm); broader (15–17 vs 10.4–12.5 µm and 2.0–2.3 vs 2.2–2.6 times as long as high) and differently shaped lip region (lips distinctly separated, strongly angular with wider expansion vs less separated, moderately angular with less expansion) and wider amphidial aperture (8.0–10 vs 5.0–7.5 µm); long and slender odontostyle (18–19 vs 11–14 µm or 6.0–7.4 vs 5.0–6.5 times as long as wide), longer odontophore (24–28 vs 16.5–18.5 µm) and total stylet length (42–46 vs 28–32.5 µm); longer pharyngeal length and its expansion (300–339 vs 210–291 µm, 129–156 vs 99–122 µm), longer cardia (17–26 vs 11–15 µm); longer tail length (35–49 vs 25.5–33 µm) and longer spicules (39–45 vs 32 µm). The new species differs from *E.pectinatus* in having a pre-rectal blind sac (vs pre-rectal blind sac absent); differently shaped tail (tail dorsally convex-conoid conoid, slightly bent ventrally with rounded terminus vs tail dorsally convex-conoid to sub digitate with acute terminus), ventral surface of the tail not pectinate (vs ventral surface of tail pectinate) and longer spicules (39–45 vs 32 µm). From *E.pussulosus* in its more posteriorly vulval position (V = 59–63 vs 48–55%); presence of pre-rectal blind sac (vs absence); smaller spicules (39–45 vs 47–49 µm), fewer ventromedian supplements (6–8 vs 17), first one beginning outside the range of spicules (vs within the range of spicules) and differently shaped tail (tail conoid, dorsally convex, straight to slightly bent ventrally vs dorsally concave, straight to slightly bent dorsally). From *E.coniceps* in its smaller body size (L = 1.0–1.3 vs 1.6–2.6 mm); differently shaped lip region (lip region offset by distinct constriction, lips angular, separated vs offset by slight depression, lips amalgamated); lower c value (23–35 vs 41–54); posteriorly vulval position (V = 59–63 vs 42–49%); presence of pre-rectal blind sac (vs blind sac absent); smaller spicules (39–45 vs 60–80 µm) and differently shaped tail terminus (tail tip rounded to sub-acute vs tail tip acute, pointed). From *E.schraederi* in having a shorter and robust body (L = 1.0–1.3 vs 1.6–2.6 mm, a = 22–30 vs 44–53), shorter tail (c’ = 1.3–1.6 vs 4.4), comparatively shorter odontostyle (18–19 vs 20 µm); posteriorly vulval position (V = 59–63 vs 51%); presence of pre-rectal blind sac (vs absence) and presence of male (vs male absent).

In the presence of blisters on the ventral cuticle of the tail, the new species is also similar to *E.maritus* Andrássy, 1959 but differs in having a shorter and robust body (L = 1.0–1.3 vs 2.0–2.3 mm, a = 22–30 vs 30–37), narrower lip region offset by constriction (15–17 vs 19–21 µm, offset by depression), shorter odontostyle (18–19 vs 24–28 µm); lower b (3.5–3.9 vs 4.0–5.0) and c (23–35 vs 40–43) values, and posterior vulval position (V = 59–63 vs 48–50%).

In its medium-sized body and the presence of pre-rectal blind sac, the new species is similar to *E.productus* (Thorne & Swanger, 1936) Andrássy, 1959 and *E.bombilectus* Andrássy, 1962 but differs from former in having a slender body (a = 22–30 vs 18.9–22.6), comparatively wider lip region offset by constriction (15–17 vs 13–14.5 µm, lip region continuous or offset by slight depression); longer odontostyle (18–19 vs 14–15 µm) and odontophore (24–28 vs 19–24 µm); comparatively posterior vulval position (V = 59–63 vs 52–55%); presence of blisters on the ventral cuticle of the tail (vs blisters absent) and comparatively smaller spicules (39–45 vs 45–50 µm).

The new species differs from *E.bombilectus* in its lip region offset by constriction (vs depression), longer odontostyle (18–19 vs 14 µm); comparatively lower b value (3.5–3.9 vs 4.2–4.4) in female; comparatively posterior vulval position (V = 59–63 vs 52–54%), cuticle near vulva simple, without any sclerotization (vs finely wrinkled near vulva); presence of blisters on the ventral side of the tail (vs blisters absent) and fewer numbers of ventromedian supplements (6–8 vs 10–12).

### ﻿ Eudorylaimus saccatus sp. nov.

Taxon classificationAnimaliaDorylaimidaDorylaimidae

C35C57A4-B9F1-522A-AB42-04050FE75405

https://zoobank.org/3E86F85B-C7F3-40C4-96F0-949FD1476BB4

**Figure 7.**
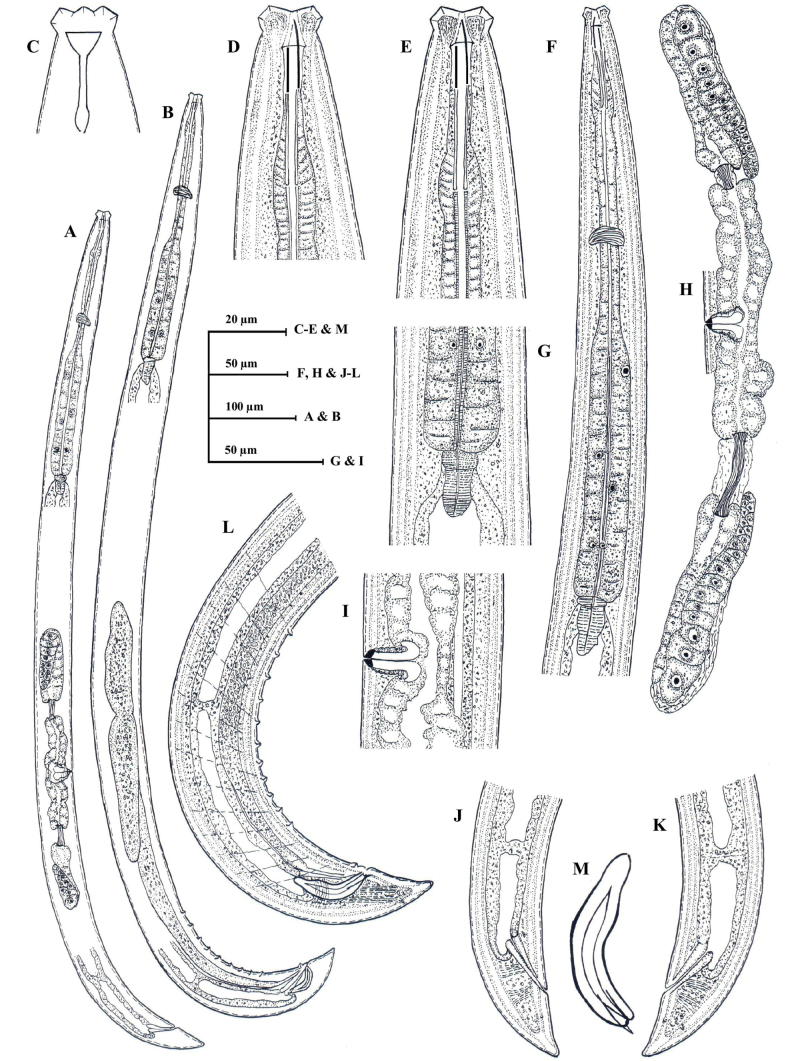
*Eudorylaimussaccatus* sp. nov. **A** entire female **B** entire male **C** anterior region showing amphid **D, E** anterior region **F** pharyngeal region **G** pharyngo-intestinal junction **H** female genital system **I** vulval region **J, K** female posterior region **L** male posterior region **M** spicule.

**Figure 8.**
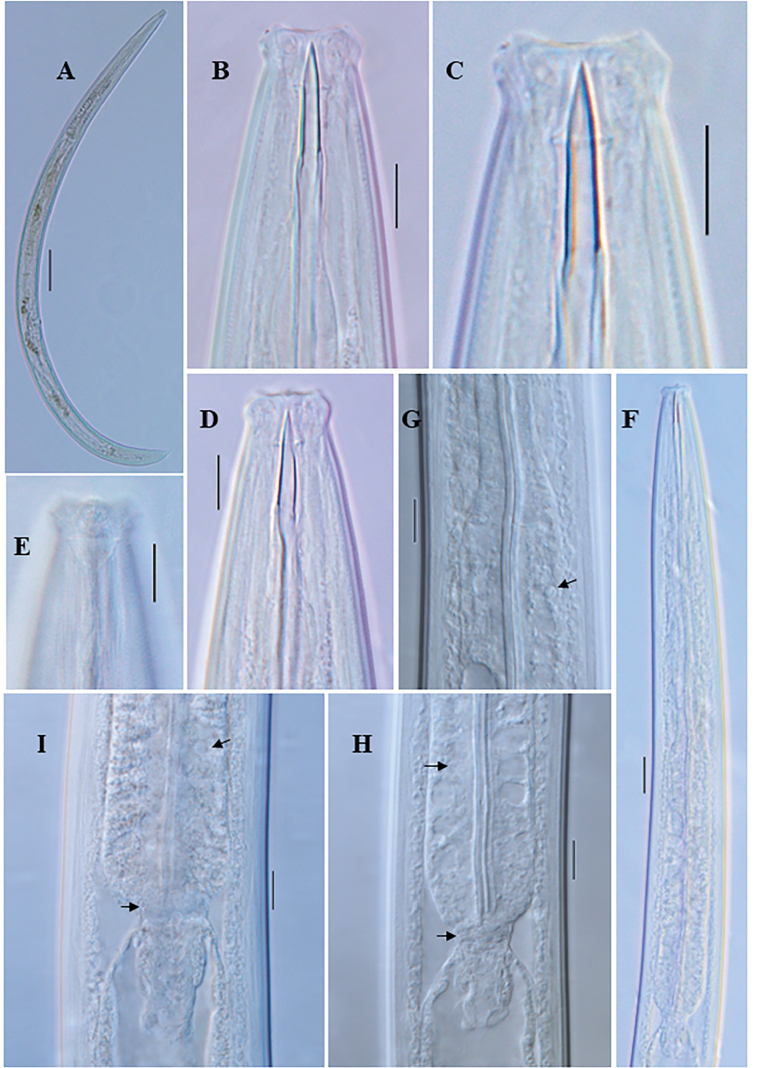
*Eudorylaimussaccatus* sp. nov. female: **A** entire **B–D** anterior region **E** anterior region showing amphid **F** pharyngeal region **G** pharyngeal expansion (arrow showing DN) **H, I** pharyngo-intestinal junction (arrow showing S2N and cardiac disc). Scale bars: 100 µm (**A**); 10 µm (**B–E**, **G–I**); 20 µm (**F**).

**Figure 9.**
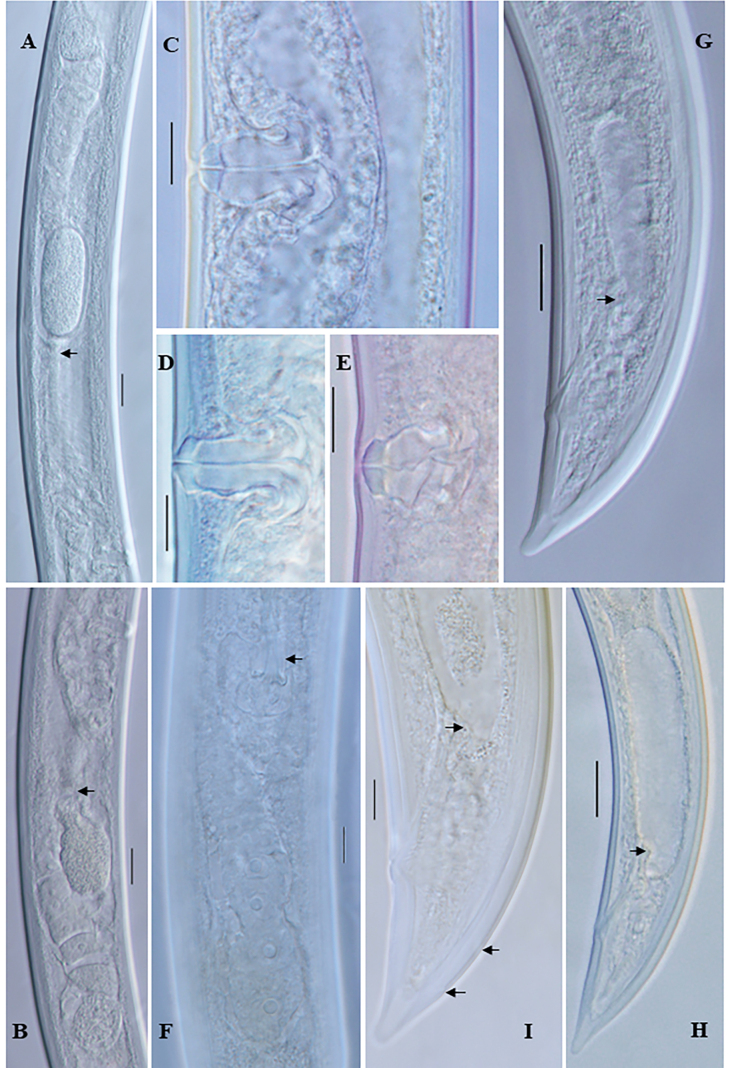
*Eudorylaimussaccatus* sp. nov. female: **A, B** genital system (arrow showing sphincter) **C–E** vulval region **F** oviduct and ovary (arrow showing sphincter) **G, H**. posterior region (arrow showing blind sac) **I** posterior end (arrow showing blind sac and caudal pores). Scale bars: 20 µm (**A, B, G, H**); 10 µm (**C–F, I**).

**Figure 10.**
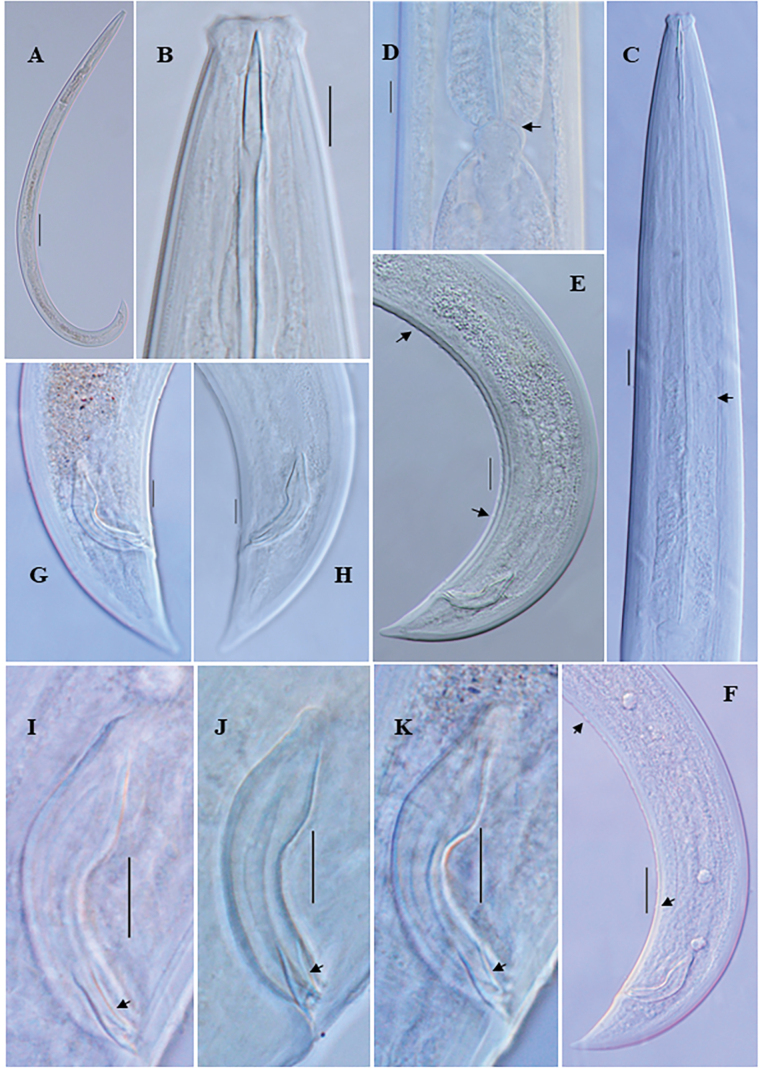
*Eudorylaimussaccatus* sp. nov. male: **A** entire **B** anterior region **C** pharyngeal region **D** pharyngo-intestinal junction (arrow showing cardiac disc) **E, F** posterior region (arrow showing series of supplements) **G, H** posterior end **I–K** spicules. Scale bars: 100 µm (**A**); 10 µm (**B, D, G–K**); 20 µm (**C, E, F**).

Figs 7 , 8 , 9 , 10 , Tables 5 , 6

#### Type material.

***Holotype*** • China (IAE/NC/EU/*E.saccatus* /1), Liaoning Province, Dalian City, Wafangdian; 39.584857°N, 121.803631°E; soil samples collected from around the roots of grasses (unidentified). ***Paratypes*** • China (4♀/5♂; IAE/NC/EU/*E.saccatus*/2-9), same data as holotype.

#### Diagnosis.

*Eudorylaimussaccatus* sp. nov. is characterized by its 1.1–1.4 mm long slender body; lip region offset by constriction, 15–17.5 μm broad; odontostyle 17.5–18.5 μm with an aperture ~ 37–45% of its length, odontophore 24.5–27.5 μm, 1.3–1.5 times the odontostyle length, total stylet length 42–45.5 μm; pharynx 332–389 μm long, pharyngeal expansion 148–189 μm or ~ 44–48% of the total neck length; cardia long 23–37 μm or 0.48–0.67 times corresponding diameter long; female genital system didelphic-amphidelphic, uterus long, well-differentiated; tail short (35–49 μm, c = 24–39, c’ = 1.0–1.6), dorsally convex, conoid, with rounded to sub-acute terminus, hyaline part 21–31% of its length; males with 44–48 µm long spicules, lateral guiding pieces distinct, rod-like, slightly curved ventrally, bifurcated with claw-like distal end and 9–11 irregularly spaced ventromedian supplements with hiatus.

**Table 5.** Morphometrics of *Eudorylaimussaccatus* sp. nov. All measurements are in μm and in the form: mean ± s.d. (range).

Characters	Holotype female	Paratype Females	Paratype Males
n		4	5
L	1334	1270.7 ± 110.4(1140–1412)	1326.8 ± 91.5 (1151–1412)
Body diameter at neck base	53	49.7 ± 6.1(42–58)	57.7 ± 6.6(50.5–67)
Body diameter at mid body	60	55.6 ± 6.0(48–63)	64.2 ± 8.9(53–76)
Body diameter at anus	33.5	32.2 ± 2.8(29–36)	33.0 ± 1.8(30–35)
a	22.2	22.9 ± 0.056(22–24)	20.9 ± 1.8(18–24)
b	3.6	3.4 ± 0.13(3.3–3.6)	3.8 ± 0.19(3.4–3.9)
c	29	26.7 ± 2.2(24–30)	33.5 ± 3.5(29–39)
c’	1.3	1.4 ± 0.12(1.3–1.6)	1.2 ± 0.12(1.0–1.3)
V	61.9	62.5 ± 0.55(61.9–63.3)	–
G1	20.6	17.3 ± 1.3(15–19)	–
G2	17.3	15.6 ± 0.78(14–17)	–
Lip region diameter	16	15.7 ± 0.43(15–16)	16.2 ± 0.67(15.5–17.5)
Lip region height	6.5	6.3 ± 0.21(6.0–6.5)	6.3 ± 0.24(6.0–6.5)
Amphidial aperture	8.5	8.2 ± 0.25(8.0–8.5)	9.0 ± 0.63(8.5–10)
Odontostyle length	17.5	18.1 ± 0.21(18.0–18.5)	18.0 ± 0.31(17.5–18.5)
Odontophore length	26	26.2 ± 0.90(25.0–27.5)	25.8 ± 0.81(24.5–27)
Total stylet length	43.5	44.3 ± 0.73(43.5–45.5)	43.8 ± 1.0(42–45)
Guiding ring from anterior end	9.0	9.3 ± 0.41(9.0–10)	8.7 ± 0.50(8.0–9.5)
Nerve ring from anterior end	136	136.2 ± 6.4(130–145)	136.0 ± 6.5(126–145)
Neck length	367	366.2 ± 17.8(342–389)	344.8 ± 10.3(332–362)
Expanded part of pharynx	176	172 ± 14.1(157–189)	156 ± 6.2(148–165)
Cardia length	28	30 ± 6.04(23–36)	30.4 ± 5.0(28–37)
Anterior genital branch	276	226.6 ± 32.7(181–256)	–
Posterior genital branch	232	203.6 ± 27.7(167–234)	–
Vaginal length	23	24.6 ± 1.6(23–27)	–
Vulva from anterior end	826	795.2 ± 71.2(717–898)	–
Prerectum length	82	75.7 ± 9.2(61–84)	111.4 ± 20.2(86–141)
Rectum length	39	33.5 ± 4.7(28–40)	43.6 ± 3.9(38–48)
Tail length	46	47.5 ± 0.86(47–49)	39.8 ± 3.0(35–44)
Spicules length	–	–	45.8 ± 1.3(44–48)
Lateral guiding pieces	–	–	13.1 ± 0.91(12–14)
Ventromedian supplements	–	–	9–11

#### Description.

**Female.** Slender nematodes of small-size, 1.1–1.4 mm long body; curved ventrally or open C-shaped upon fixation. Body cylindrical, tapering gradually towards both extremities but more so towards the posterior region. Cuticle with two distinct layers, 1.5–2.0 μm thick at the anterior region, 3.0–4.0 μm at midbody, and 5.0–6.0 μm on tail. Outer cuticle smooth, inner layer thin with distinctly fine transverse striations. Lateral, dorsal and ventral body pores indistinct. Lateral chords 11–14 μm wide at midbody, occupying ~ 1/5 (20–22%) of the corresponding body diameter. Lip region offset from the body by constriction, 2.4–2.5 times as wide as high or ~ 1/3 (27–35%) of the body diameter at the neck base. Lips angular, separated. Amphidial fovea funnel-shaped, aperture slit-like, 8.0–8.5 μm wide or occupying ~ 1/2 (50–53%) of lip region diameter. Cheilostom a truncate cone. Odontostyle typical dorylaimoid, 5.8–6.1 times as long as wide, 1.1–1.2 times the lip region diameter long or 1.2–1.5% of total body length, its aperture 7.0–8.5 μm or ~ 2/5 to 1/2 (38–47%) of its length. Odontophore linear, rod-like, 1.3–1.5 times the odontostyle length. Guiding ring simple, at 0.56–0.62 times lip region diameter from the anterior end. Pharynx consisting of a weakly muscular anterior part, expanding gradually into a cylindroid part, occupying ~ 44–48% of the total neck length, expanded part of the pharynx 5.7–6.7 times as long as wide, 3.2–3.7 times body diameter at neck base. Pharyngeal gland nuclei and their orifices are located as follows: DO = 56.2–58.4, DN = 60.3–63.4, DO–DN = 3.1–4.9, S1N1 = 74.2–77.8, S1N2 = 80.8–83.8, S2N = 89.4–90.9, S2O = 90.3–92.2. Nerve ring encircling the pharynx at 36–38% of pharyngeal length from the anterior end. Cardia long, rounded to conoid, 1.5– to 2.2 times as long as wide or ~ 1/2 to 2/3 (52–67%) of the corresponding body diameter long, its junction with pharyngeal base apparently surrounded by cardiac disc (Fig. 8H, I). Genital system didelphic–amphidelphic; both the genital branches almost equally developed. Anterior genital branch 15.8–20.6% and the posterior branch 14.6–17.3% of body length. Ovaries reflexed, not reaching the oviduct-uterus junction; measuring 75–170 μm or 1.5–2.6 (anterior) and 85–107 μm or 1.3–1.7 (posterior) times the corresponding body diameter long; oocytes arranged in a single row except near the distal end. Oviduct joining the ovaries sub-terminally, measuring 86–190 μm or 1.7–3.0 (anterior, with one uterine egg inside, egg ovoid, 75 × 36 μm, 2.0 times as long as wide) and 75–136 μm or 1.5–2.1 (posterior) times the corresponding body diameter long; consisting of a slender distal portion and a well-developed ***pars dilatata***. Oviduct-uterus junction marked by a well-developed sphincter. Uterus well-differentiated, tripartite, the proximal part well-developed muscular, the median short, comparatively less muscular, and the distal part long, tube-like, somewhat spheroid at the end, measuring 96–133 μm or 2.0–2.1 (anterior,) and 85–107 μm or 1.3–1.7 (posterior) times the corresponding body diameter long; Vagina extending inwards, 23–27 μm or ~ 2/5 to 1/2 (38–47%) midbody diameter; ***pars proximalis*** 15–21 × 11–12 μm, with somewhat sigmoid wall encircled by circular muscles; ***pars refringens*** with two small trapezoid-shaped sclerotized pieces, measuring 4–5 × 4–5 μm and the combined width 8.5–10 μm; ***pars distalis*** 1.5–2.0 μm. Vulva a transverse slit. Sperm cells present (*n* = 3). Prerectum 1.7–2.8 and rectum 0.83–1.2 times the anal body diameter long, a distinct blind sac extending posteriorly to the prerectum-rectum junction (Fig. 9G–I). Tail dorsally convex conoid, its distal end straight to slightly bent ventrally with a rounded to sub-acute terminus, 1.3–1.6 times anal body diameter long; the hyaline part of the tail always perceptible, ~ 1/5to 1/3 anal body diameter long with a pair of caudal pores on dorsal side.

**Table 6.** Some important morphometrics and morphological characters of *Eudorylaimussaccatus* sp. nov. and its six close species. All measurements are in um (except for ‘L’ in mm).

Character	*Eudorylaimussaccatus* sp. nov.	*E.productus* Andrássy, 1959	*E.bombilectus* Andrássy, 1962	*E.blisterocaudatus* sp. nov.	*E.opisthohystera* Altherr, 1953	*E.subdigitalis* Tjepkema et al., 1971	*E.piecea* Wu et al., 2018
*n*	5♀♀/5 ♂♂	13♀♀/1♂	?♀♀/?♂♂	7♀♀/4 ♂♂	?♀♀/?♂♂	15♀♀	7♀♀
L	1.1–1.4/1.1–1.4	1.0–1.1/1.0–1.2	1.3–1.7/1.0	1.0–1.3/1.0–1.1	1.3–1.5/1.5	14–1.5	1.0–1.2
a	22–24/18–24	18–22/20–24	21–25/20–23	22.0–29.9/24.6–26.5	22–29/33	20.5–27.3	15–21.6
b	3.3–3.6/3.4–3.9	3.5–4.1/3.5–4.0	4.2–4.4/3.6–3.8	3.5–3.9/3.4–3.6	3.2–3.6/4.3	3.0–3.8	3.1–3.6
c	24–30/29–39	28–33/30–34	28–38/33–37	23.3–34.7/28.3–33.0	22–29/30	26–40	18.7–27.5
c’	1.3–1.6/1.0–1.3	1.1–1.5/1.1–1.2	1.3–1.5	1.3–1.7/1.2–1.3	–	–	1.5–1.8
Vulval position (V%)	61.9–63.3	52–54	52–54	59.1–63.2	61–64	56.5– 60.9	59.5–61.8
Lip region	offset by constriction	almost continuous	offset by depression	offset by constriction	offset by constriction	moderately offset by constriction	offset by constriction
Lips shape	Lips moderately angular, separated	Lips less angular	Lips moderately angular	Lips strongly angular, well-separated	Lips strongly angular, well-separated	Lips moderately angular and separated	Lips strongly angular, well separated
Amphidial aperture	8.0–8.5/8.5–10	7.0–7.5	–	8.0–10/8.0–9.5	10	8–10	5.0–6.5
Odontostyle length	17.5–18.5/ 17.5–18.5	14–14.5/14–15	14	18–18.5/ 18–19	19.6/18	18.2	20–22
Odontophore length	25–27.5/24.5–27	19.5–24/19.0–21.5	–	24–26/24–26	25/27	–	22–25
Total stylet length	43.5–45.5/42–45	33.5–38/33–36.5	–	42–44/42–45	44.6/45	–	42–47
Neck length	342–389/332–362	273–320/281–313	–	301–339/300–332	360–400/350	310–370	560–620
Expanded part of pharynx	157–189/148–165	115–141/118–136	–	132–156/129–150	–	–	140–156
Ex. part of pharynx (%)	44–48	41–44	38–40	41–47	44/47	50	42–55
Cardia length	23–36/28–37	17.5–22	–	17–23/20–23	25–40	16–33	13–20
Prerectum length	61–84/86–141	50–69/64–89	–	37–85/58–128	81	60	72–107
Tail length	46–49/35–44	33–41/34–38	–	37–49/35–39	–	–	46–59
Tail shape	Conoid, tip rounded to sub-acute terminus	Conical, tip finely rounded	Conical, tip pointed	Conoid, tip rounded	Conoid, tip rounded	Conoid, digited tip	Conoid, tip rounded
Spicules	44–48	45–50	43–45	39–42	55	–	–
Ventromedian supplements	9–11	7–10	10–12	6–8	12	–	–
Blisters on tail	Absent	Absent	Absent	Present	Absent	Absent	Absent
Pre-rectal blind sac	Present	Present	Present	Present	Absent	Absent	Absent

**Male.** General morphology similar to that of female except for the posterior region being more ventrally curved. Genital system diorchic, testes opposed, sperm cells spindle-shaped, 5.0–7.5 μm long. In addition to the adcloacal pair, situated at 7–8 µm from the cloacal aperture, there are 9–11 irregularly spaced ventromedian supplements, the first ventromedian supplement located outside the range of spicules, 44–48 μm from the adcloacal pair. Spicules typically dorylaimoid, curved ventrad, relatively robust, 4.0–4.6 times as long as wide and 1.2–1.5 times as long as body diameter at the level of cloacal aperture, dorsal contour regularly convex, ventral contour bearing a moderately developed hump, curvature of 124–130°. Head occupying 16.6% of total spicules length, its dorsal side longer than ventral side, both sides slightly curved. Median pieces 10.6–12.3 times as long as wide or occupying ~ 27.2–35% of the spicules’ maximum width, reaching the spicules tip, posterior end 3.5–4.0 μm wide. Lateral guiding pieces distinct, rod-like, slightly curved ventrally, bifurcated with claw-like distal end, ~ 4.8–5.6 times as long as wide or ~ 1/4 to 1/3 of the spicules’ length. Prerectum 2.6–4.4 and rectum 1.0–1.5 cloacal body diameter long. Tail dorsally convex, conoid, tapering gradually, its distal end slightly bent ventrally with rounded to sub-acute terminus, 1.0–1.3 times cloacal body diameter long, the hyaline part of the tail always perceptible, ~ 1/4 to 1/3 anal body diameter long with a pair of caudal pores on dorsal side.

#### Etymology.

The new species is named *Eudorylaimussaccatus* because of the pre-rectal blind sac.

#### Remarks.

In the presence pre-rectal blind sac, the new species is similar to *E.productus*; *E.bombilectus* Andrássy, 1962 and *E.blisterocaudatus* sp. nov., but differs from the former in having comparatively broader and differently shaped lip region (15–17.5 vs 13–15 µm, lip region offset by constriction, lips more angular vs lip region almost continuous, lip comparatively less angular); longer and broader odontostyle (17.5–18.5 vs 14–15 µm, 5.8–6.1 vs 6.3–7.5 times as long as wide), comparatively longer odontophore and total stylet (24.5–27.5 vs 19.5–24.0; 43.5–45.5 vs 33.5–38.5 µm); long and slender expanded part of pharynx (148–189 vs 115–141 µm or 5.7–6.7 µm vs 3.7–5.0 times as long as wide), anteriorly located dorsal pharyngeal gland nuclei (DN = 60–63 vs 63–68); more posterior vulval position (V = 61–63 vs 52–54%) and differently shaped tail (tail dorsally convex, conoid, straight to continuously curved ventrad vs tail conoid, first ventrally straight two-thirds then curved ventrad). The new species differs from *E.bombilectus* in having a comparatively shorter body size (L = 1.1–1.4 vs 1.3–1.7 mm); longer odontostyle (17.5–18.5 vs 14 µm) and expanded part of pharynx (44–48 vs 38–40% of the total pharyngeal length); more posteriorly vulval position (V = 61–63 vs 52–54%) and differently shaped tail terminus (tail tip rounded to sub-acute vs tail tip acute, pointed). From *E.blisterocaudatus* sp. nov. in having differently shaped lip region (lips separated, moderately angular vs lips well-separated, strongly angular) and amphid (funnel-shaped vs cup-shaped); comparatively longer pharynx and its expanded part (332–389 vs 300–339 µm, 148–189 vs 129–156 µm); longer spicules (44–48 vs 39–45 µm) and more ventromedian supplements (9–11 vs 6–8); differently shaped tail (tail conoid with rounded to subacute terminus vs tail conoid with sub-clavate to rounded terminus) and absence of blisters on the ventral side of the tail (vs blisters present).

Based on the body size and pattern of the tail the new species also resembles *E.opisthohystera* Altherr, 1953; *E.subdigitalis*Tjepkema et al. 1971 and *E.piecea* Wu et al. 2018 but differs from the former in having comparatively shorter male body size (L = 1.1–1.4 vs 1.5 mm) and odontostyle (17.5–18.5 vs 18–20 µm); shorter tail length (35–47 vs 58–73 µm); presence of pre-rectal blind sac (vs blind sac absent); comparatively smaller spicules (44–48 vs 55) and fewer ventromedian supplements (9–11 vs 12). The new species differs from *E.subdigitalis* in having shorter pharyngeal expansion (44–48 vs 50% of pharyngeal length) and presence of cardiac disc at the pharyngo-intestinal junction (vs absent); comparatively more posterior vulval position (V = 61–63 vs 56–60), longer prerectum (61–84 vs 60 µm), presence of pre-rectal blind sac (vs blind sac absent) and presence of male (vs male not known). From *E.piecea* in its comparatively slender body (a = 22–24 vs 15–21.6) and shorter odontostyle (17.5–18.5 vs 20–22 µm); longer pharyngeal expansion (157–189 vs 140–156) and cardia (23–36 vs 13–20 µm), presence of cardiac disc at the pharyngeal-intestinal junction (vs absent); comparatively more posterior vulva position (V = 61–63 vs 59–61), presence of pre-rectal blind sac (vs absent) and presence of male (vs absent).

## ﻿Discussion

The genus *Eudorylaimus* is one of the most speciose soil-inhabiting nematode taxa. With the addition of four new species (*E.lautus*, *E.humilior*, *E.tarkonensis*, and *E.maritus*), he (loc. cit.) transferred 127 *Dorylaimus* species to *Eudorylaimus*; of these species, the single largest contribution was that of Thorne and Swanger (1936), and 36 new species of *Dorylaimus* of their monograph work were transferred to *Eudorylaimus* by Andrássy (1959). After the proposal of the genus, several authors, including Andrássy (1960, from Afghanistan; 1962a, b, 1986, 1987, and 1991 from Hungary; 1963, from Argentina; 1967, from Chile; 2008, from Antarctica; 2009 from Albania); Brzeski (1960, 1962a from Poland; 1962b, from Norway); Heyns (1963, from South Africa; 1993, from Antarctica); Lordello (1965, from Brazil); Eliava (1968, from Georgia); Yeates (1970, from Antarctica) added new species to the genus from different parts of the world. Tjepkema et al. (1971) while revising the genus *Eudorylaimus* added ten new species, redescribed several known species from Indiana, and restudied the type material of some species. Until that time the genus already contained a great number of species, increasing up to 170, so it was not easy to identify specimens to species level. Thus, of these, they divided only 101 species of the genus into six species groups (*carteri*, *humulis*, *lugdunensis*, *miser*, *granuliferus*, and *nothus*) to make it easier for species identification based on lip region morphology, the nature of the odontostyle, its width and length, and tail morphology. However, Andrássy (1986) did not accept their division of the genus due to the lack of distinguishable characters; moreover, based on tail shape, he divided all the species into two broad groups: the first group contained the conoid tailed species, and the second group contained round tailed species and later, the round tailed species were transferred to *Thonus* Thorne, 1974. Simultaneously, he (loc. cit.) proposed three more new genera to accommodate some of the species transferred to *Eudorylaimus* from *Dorylaimus*, i.e., *Epidorylaimus* for *Eudorylaimuslugdunensis* (*Dorylaimuslugdunensis* de Man,1880) and species of this newly proposed genus were identical to species of the *lugdunensis* group (*Allodorylaimus* for *Eudorylaimusuniformis* (*Dorylaimusuniformis* Thorne & Swanger, 1936) and *Microdorylaimus* for *Eudorylaimusparvus* (*Dorylaimusparvus* de Man, 1880)). Andrássy (1986) also provided a list of species transferred to other genera (*Afrodorylaimus*, *Aporcelaimellus*, *Aporcelaimus*, *Aquatides*, *Chrysonemoides*, *Discolaimium*, *Discolaimoides*, *Dorydorella*, *Ecumenicus*, *Labronema*, *Laimydorus*, *Longidorella*, *Oriverutus*, *Paramonovia*, *Pungentus*, *Rhyssocolpus*, *Thonus*, and *Willinema*). Finally, he (loc. cit.) revised the diagnosis of the genus *Eudorylaimus*, provided a list of 58 valid species till that time, and key to species for the first time. Later, several authors (Zell 1986; Andrássy 1991; Khan 1989) added more new species to the genus from different countries and transferred several species to other genera. Therefore, the number of valid species increased from 58 to 80 and Andrássy (1991) also added a key to the remaining 22 species of the genus described between 1986 and 1991. In his monographic work, Andrássy (2009a) provided a list of valid species of this taxon. During the last few decades, several authors (Kito et al. 1996, from Antarctica; Vinciguerra and Orselli 1998, from Italy; Khan and Araki 2001 and Ahmad and Araki 2003 from Japan; Mushtaq and Ahmad 2006 from India; Gagarin and Naumova 2011, 2012 from Russia; Moslehi et al. 2012 and Kazemi et al. 2018 from Iran; and Gusakov et al. 2022 from Russia) added several new species to the genus. The representatives of the genus *Eudorylaimus* are widely distributed and recorded from all the continents and, interestingly, more than ten species have also been recorded from Antarctica (Loof 1975; Heyns 1993; Kito et al. 1996; Andrássy 2008; Velasco-Castrillon and Stevens 2014; Elshishka et al. 2023; Holovachov 2014).

The species of the *Eudorylaimus* can be distinguished from each other by the body size, shape of lip region (offset by deep constriction or lip region continuous; lips separated, more elevated and strongly angular or lips amalgamated, moderately elevated and angular); size and shape of odontostyle; length of expanded part of pharynx; presence or absence of cardiac disc; the position of the vulva (%) from the anterior end; the shape of vulval opening (transverse, pore-like or longitudinal); presence or absence of vulval papillae; shape of *pars refringens* and uterus; size and shape of tail; presence or absence of blister on the tail cuticle; shape and size of spicules and the number of ventromedian supplements are the most distinguishable characters. Despite its global distribution, the vast majority of *Eudorylaimus* species (almost 80%) are only found in the Palaearctic region (Andrássy 1986). The genus *Eudorylaimus* is one of the most dominant omnivorous dorylaimid taxa, and except marine water, its representatives have also been recorded from almost every sort of habitat such as croplands (maize, soybean, wheat, pasture, etc.), grassland, moss, lichens and microbial mat (Maslen and Convey 2006), different forests (oak, spruce, meadow, birch, pine, etc.), fallow field, meadows, moorland, freshwater (from lakes by Gagarin and Naumova 2011, 2012; Gusakov et al. 2022), extreme cold habitat (11 species from Antarctica by Velasco-Casteillon and Stevens 2014). Moreover, 12 species of the genus *Eudorylaimus* have been recorded from Polish pine forests on dunes (Wasilewska 1970), 14 species from Indiana hardwood forests (Johnson et al. 1972), 11 species from Yugoslavian dune vegetation (Krnjaic 1998), five species from Bulgarian grasslands (Eliava 1998), and 13 species on moss and lichens in the maritime Antarctic (Maslen and Convey 2006). The genus exhibits exceptional adaptions to harsh environments, such as temperate regions devoid of vegetation and Antarctic ecosystems, and its representatives tend to reach the greatest abundance at the later stage of succession in old-growth forests (McSorley 2012). The genus makes up a great majority of omnivorous nematofauna in Swedish tundra soil, and it has also been recorded from Israel’s Negev desert (Liang et al. 2002) as well as from the soil crusts of Utah (Darby et al. 2007). *Eudorylaimus* is a dominant omnivore in a large field of sandy soil without vegetation in Florida (McSorley 2011) and it also inhabits the moss growing on the tree trunks in an oak forest in Bulgaria (Lazarova et al. 2004). However, the information mentioned above reveals great habitat diversity, extremities, species richness, and global distribution of the genus.

Very few molecular data of dorylaimid nematode taxa are available in the NCBI. For the *Eudorylaimus*, 85 nucleotide (20 of 28S rDNA and 65 of 18S rDNA) sequences are available, among them 65 sequences representing 13 species have been identified (*E.altherri*, *E.caloradensis*, *E.carteri*, *E.centerocircus*, *E.conicaudatus*, *E.coniceps*, *E.meridionalis*, *E.minutus*, *E.piceae*, *E.silvaticus*, *E.sodakus*, *E.Subdigitalis*), and 21 sequences have been identified only as *Eudorylaimus* sp. Despite the 85 nucleotide sequences, only one species, *E.piceae*, has also been described based on molecular taxonomic (Wu et al. 2018a) from China.

To date, more than 111 species have been recorded from all over the world but only one species is recorded from China (Peña-Santiago 2021; Wu et al. 2018a) so far. Although Andrássy (1986, 1991, 2009a) tried to make this group more homogenous; however, in the last four decades with the addition of a large number of new species, this genus has again become very difficult to handle while identifying any representative. Hence, a thorough revision of the genus based on the study of the types is needed to provide detailed morphology, molecular barcoding, phylogeny, variability, and geographical distribution.

## Supplementary Material

XML Treatment for Eudorylaimus meridionalis

XML Treatment for Eudorylaimus caudatus

XML Treatment for Eudorylaimus blisterocaudatus

XML Treatment for Eudorylaimus saccatus

## ﻿Additional information

### Author ORCIDs

Md Niraul Islam https://orcid.org/0000-0001-8520-9775

Ying bin Li https://orcid.org/0000-0002-0207-8667

Wasim Ahmad https://orcid.org/0000-0002-8545-4997

Saleh Alhewairini https://orcid.org/0000-0003-2770-7412

Qi Li https://orcid.org/0000-0002-8652-8396

### Data availability

All of the data that support the findings of this study are available in the main text.
